# Elevation of ISG15 promotes diabetic kidney disease by modulating renal tubular epithelial cell pyroptosis

**DOI:** 10.1002/ctm2.70337

**Published:** 2025-06-03

**Authors:** Lingzhi Huang, Xinyi Chen, Yawen Shao, Shujun Deng, Chen Wang, Jianqiao Chen, Yongsheng Xie, Siming Yuan, Liqin Tang

**Affiliations:** ^1^ Department of Pharmacy The First Affiliated Hospital of USTC Division of Life Sciences and Medicine University of Science and Technology of China Hefei Anhui China; ^2^ Anhui Provincial Key Laboratory of Precision Pharmaceutical Preparations and Clinical Pharmacy The First Affiliated Hospital of USTC Hefei Anhui China

**Keywords:** diabetic kidney disease, fibrosis, ISG15, pyroptosis, renal tubular epithelial cells

## Abstract

**Background:**

Fibrosis and inflammation in the renal tubular epithelial cells (TECs) are key contributors to the pathology of diabetic kidney disease (DKD). Nevertheless, the precise triggers of these processes remain unclear. This study aimed to explore the role of interferon‐stimulated gene 15 (ISG15) in the injury of TECs induced by high glucose (HG) conditions and its implications for the development of DKD.

**Methods:**

ISG15 knockout (ISG15 KO) mice injected with streptozotocin‐treated mice on a high‐fat diet were used to investigate its role in DKD. Cellular models with ISG15 knockdown were exposed to HG conditions to assess the effects of ISG15 on cellular responses. Subsequently, we evaluated the impact of ISG15 on pyroptosis, a form of programmed cell death, to understand its potential role in DKD pathology. Furthermore, RNA sequencing (RNA‐seq) and molecular biology techniques were employed to explore the signalling pathways potentially regulated by ISG15.

**Results:**

We first confirmed an up‐regulation of ISG15 within the renal tubule in DKD. The deletion of ISG15 alleviated renal functional damage, fibrosis and inflammation, which correlated with reduced ISGylation levels. Mechanistic investigation revealed that HG stimulation in TECs disrupted the mtDNA–cGAS–STING signalling, which exacerbates the DKD through the NLRP3–CASP1–GSDMD axis. Furthermore, we uncovered a bidirectional regulatory loop between STING and ISG15, with STING enhancing ISG15 expression upstream and ISG15 modulating STING expression through ISGylation.

**Conclusion:**

ISG15–mtDNA–STING emerges as a critical hub that integrates the processes of pyroptosis, fibrosis and inflammation. Therapeutic interventions that target this signalling network at various levels may pave the way for innovative treatments for DKD.

**Key points:**

- ISG15 is highly expressed in both DKD mice and renal tubular epithelial cell cultured in HG condition.
- ISG15 promotes DKD pyroptosis via NLRP3–CASP1–GSDMD axis.
- ISG15–mtDNA–STING emerges as a critical hub that integrates the processes of pyroptosis.

## Graphical abstract

1. ISG15 is highly expressed in both DKD mice and renal tubular epithelial cell cultured in HG condition.
2. ISG15 promotes DKD pyroptosis via NLRP3–CASP1–GSDMD axis.
3. ISG15–mtDNA–STING emerges as a critical hub that integrates the processes of pyroptosis

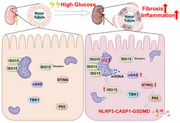

## 1 INTRODUCTION

Diabetic kidney disease (DKD), alternatively referred to as diabetic nephropathy, is a prevalent chronic complication of Type 1 and Type 2 diabetes with albuminuria and progressive renal failure.^1^ Hyperglycaemia can trigger a variety of intracellular processes that promote renal fibrosis and inflammation, predominantly through pathways involving fatty acid metabolism and oxidative stress.^2^ It is estimated that approximately 20–40% of patients with diabetes will develop some form of kidney disease, representing a significant public health challenge.^3^ Recent studies highlight the pivotal role of renal tubular damage in DKD progression.^4^, ^5^ Moreover, patients with DKD experience a range of structural changes in their renal tubules, including renal tubular atrophy and interstitial fibrosis, which are closely associated with renal damage.^6^ On a cellular level, mitochondria – the organelles responsible for energy production – are key regulators of renal tubular damage in DKD. There is growing evidence that renal tubular mitochondrial dysfunction is associated with renal tubular injury.^7^, ^8^ Despite the complex nature of DKD, the precise mechanisms that initiate renal tubular epithelial cell (TEC) injury in DKD are not fully understood. Current treatment options for DKD are limited and often inadequate for a significant number of patients. This has prompted researchers to search for novel therapeutic targets and to develop innovative strategies aimed at improving treatment outcomes for those affected by the disease.

Interferon‐stimulated gene 15 (ISG15), a critical component of the IFN‐I response, is recognised as the first discovered ubiquitin‐like protein.^9^, ^10^ Its principal role is to mediate the covalent attachment to lysine residues on target proteins through a series of enzymatic reactions, a process termed ISGylation.^11^, ^12^, ^13^ This post‐translational modification is increasingly recognised for its key role in modulating both adaptive and innate immune responses.^14^, ^15^, ^16^ Additionally, ISG15 can exert its effects in a non‐covalent, free form.^17^, ^18^ It is indicated by research that ISG15 engages in a variety of biological processes, operating in a manner specific to cells and tissues. For instance, within the realm of cancer biology, ISG15/ISGylation has been implicated in reinforcing ATG7 stability, thereby enhancing autophagy in pancreatic cancer cells – a mechanism that fosters tumour progression and therapeutic resistance.^19^ Similarly, in glioma, ISG15 bolsters Oct4 stability, positively regulating stemness properties of these malignant cells.^20^ Conversely, aberrant activation of the ISG15/ISGylation pathway has been associated with promoting cancer cell proliferation, migration and invasion, underscoring its dualistic nature in oncogenesis. In oesophageal adenocarcinomas, ISG15 further contributes to immunosuppression by stabilising the GRAIL1 protein through ISGylation, leading to CD3 degradation and subsequent dampening of T‐cell activity, which exacerbates the disease's severity and prognosis.^21^ The implications of ISG15 dysregulation extend beyond carcinogenesis, touching upon viral pathogenesis and neurodegeneration, making it a potential linchpin in understanding and treating a spectrum of devastating diseases. Although recent findings have shed light on ISG15's cellular functions and the molecular mechanisms by which it operates, its specific role in DKD remains to be fully elucidated.

This investigation aimed to explore the impact of ISG15 on renal tubular injury and inflammation during DKD. We utilised both in vivo DKD mouse models and in vitro cellular systems to replicate the pathological conditions of DKD. Here, we found the elevated ISG15 expression in the kidneys of DKD mice. Knockout of ISG15 significantly alleviates renal functional damage, fibrosis and inflammation. Furthermore, we demonstrated a novel mechanism by which ISG15, through the process of ISGylation, regulates pyroptosis in renal TECs via mitochondrial DNA (mtDNA)‐mediated cGAS–STING signalling. Collectively, our findings establish ISG15 as a key mediator of diabetic kidney injury and propose its therapeutic targeting for DKD intervention.

## 2 MATERIALS AND METHODS

### 2.1 Animals

The ISG15 global knockout (ISG15‐KO) mouse model, established on a C57BL/6 genetic background, was commercially obtained from Cyagen Biosciences (Guangzhou, China). All mice were maintained under controlled environmental conditions, including a 12‐h light/dark cycle and constant temperature of 25 ± 1°C, with free access to autoclaved water and standard laboratory chow throughout the study period. After 4 weeks of high‐fat diet (HFD; 60% fat calories) feeding, the mice received 5 days of daily intraperitoneal streptozotocin (STZ) injections (60 mg/kg), while maintaining the HFD. The timeline for the development of DKD in the mice started from the end of the injection period, with mice euthanised for sample collection at 8 and 12 weeks for subsequent analysis. Additionally, diabetic db/db mice with C57BLKS/JGpt background and their non‐diabetic db/m littermates were obtained from GemPharmatech (Nanjing, China). They were maintained on a standard chow diet until they reached the ages of 16 and 24 weeks. Animal experiments were performed in compliance with the guidelines and protocols approved by the Animal Ethics Committee of the First Affiliated Hospital of the University of Science and Technology of China (2022‐N (A)‐012).

### 2.2 Cell cultures and treatment

Mouse glomerular mesangial cell line, glomerular endothelial cell line and podocyte cell line were obtained from Procell Technology. Mouse TEC was obtained from BeNa Culture Collection and cultured in DMEM/F12 medium (Biosharp) supplemented with 5% foetal bovine serum (Viva cell, China). To establish the HG model, cells were treated with complete culture medium containing 40 mM d‐glucose (Sigma, USA) for 48 h. To control for potential osmotic effects, parallel cultures were maintained in medium supplemented with 40 mM mannitol (MA) (Sigma) under identical conditions.

Cells were transfected with negative control siRNA, target gene‐specific siRNA, target gene‐containing plasmid or empty vector control plasmid using Lipofectamine 2000 reagent (Invitrogen, USA) according to the manufacturer's protocol. The siRNAs were synthesised by TSINGKE Biotech (Beijing, China), while the plasmids were generated by miaolingbio (Wuhan, China). The sense sequence for si‐*Isg15* (mouse) is 5′‐GAAUUUGAUGUUAUGUUUAdTdT‐3′, for si‐*Gsdmd* (mouse) is 5′‐CCUCCAUGAAUGUGUGUAUTT‐3′.

To inhibit mitochondrial reactive oxygen species (mtROS), NLRP3 or STING, cells were pretreated with the mitochondria‐specific antioxidant Mito‐TEMPO (25 µM; TargetMol, China), NLRP3‐specific inhibitor MCC950 (2 µM; TargetMol) or STING‐specific inhibitor C‐176 (10 µM; TargetMol) for 1.5 h before exposure to 40 mM HG.

### 2.3 Assessment of mitochondrial parameters: mass, ROS and membrane potential

Mitochondrial mass quantification was performed through incubation with MitoTracker (1 µM; Beyotime, China) at 37°C for 30 min in complete darkness, with subsequent PBS washes prior to fluorescence detection using the CytoFLEX (Beckman, USA). For mtROS detection, cellular specimens were subjected to staining with MitoSOX Red mitochondrial superoxide indicator (5 µM; YEASEN, China) under standard culture conditions (37°C, 30 min), followed by quantitative analysis using flow cytometry. Mitochondrial membrane potential was evaluated through JC‐1 staining according to standardised protocols provided with the commercial assay kit (Beyotime).

### 2.4 CCK‐8 assay

For cell viability assessment, a cellular suspension containing 5 × 10^3^ cells/well was plated in 96‐well culture plates. Following 48‐h incubation, culture medium was replaced with 10% CCK‐8 solution (Meilunbio, China). The cellular metabolic activity was subsequently measured after 1–2 h of incubation at 37°C using the SpectraMax i3x multifunctional microplate reader (Molecular Devices, USA).

### 2.5 Detection of Annexin V/PI double‐positive cells rate

After PBS washing of harvested cell suspensions, samples were incubated with the Detection Kit (BD Biosciences, USA). In brief, cells were incubated with PI and Annexin V‐FITC at 25°C for 15 min. Then, the stained cells were examined by the flow cytometer (CytoFLEX; Beckman).

### 2.6 LDH release assay

The determination of LDH release was conducted using a colorimetric detection kit (Beyotime) according to the manufacturer's instructions. Cells were cultured within 96‐well plates for 48 h, followed by collection and centrifugation of supernatants at 400×*g* for 5 min. A volume of 120 µL of the supernatant was transferred to a new 96‐well plate, followed by the sample assay was performed immediately.

### 2.7 Adeno‐associated virus‐mediated gene transfer

For knockdown GSDMD, the 1 × 10^11^ vg of adeno‐associated virus type 9 (AAV9) carrying sh‐GSDMD or sh‐NC (GenePharma, China) was administered i.v. through the tail vein to assess its efficacy in DKD or WT male mice. After 4 weeks of recovery, the transfection effects were observed via Western blot.

### 2.8 Intracellular ROS levels

Cells were PBS‐washed, incubated with DCFH‐DA (10 µM; Beyotime) at 37°C for 15 min in the dark, then washed three times with culture medium. After that, ROS production was subsequently measured via flow cytometry (CytoFLEX; Beckman).

### 2.9 Transmission electron microscopy

Renal ultrastructure was examined by transmission electron microscopy (TEM) according to established protocols. Tissue samples were trimmed into 1 mm^3^ cubes and fixed in 2.5% glutaraldehyde. After phosphate buffer washing, specimens were post‐fixed in 1% osmium tetroxide for 30 min. Following dehydration through an ethanol gradient, tissues were embedded in epoxy resin. Ultrathin sections (50–60 nm) were obtained and contrasted with 3% uranyl acetate. Images were captured by Servicebio Technology (Wuhan, China).

### 2.10 DNA isolation and mtDNA copy number assay

The cellular suspension was partitioned into uniform aliquots for subsequent experimental procedures. One was used to extract total DNA as a normalised control for total mtDNA. A 500 µL volume of buffer solution (50 mM HEPES, 150 µM NaCl and 25 µg/mL digitonin) was used to resuspend the second aliquot, ensuring optimal sample recovery. The samples were incubated for 10 min and then centrifuged at 980×*g* for 3 min to pellet intact cells. The mitochondrial fraction was retained from the first pellet, while the cytosolic supernatant was transferred and centrifuged at 17 000×*g* for 10 min to remove residual debris. The cell pellet was resuspended in 400 µL of chilled lysis buffer (1% NP40, 150 mM NaCl, 50 mM HEPES), incubated on ice for 30 min and centrifuged at 7000×*g* to collect the mitochondrial‐containing supernatant. The TIANamp Genomic DNA Kit (TIANGEN, China) was used for DNA extraction, and mtDNA levels were measured by quantitative polymerase chain reaction (qPCR) using mtDNA‐specific primers.

### 2.11 RNA extraction and real‐time qPCR

RNA extraction from cultured cells and renal tissues was performed with the HiPure Tissue RNA Mini Kit (Magen, China), followed by reverse transcription into cDNA. QPCR analysis was performed using SYBR Green reagent (Vazyme, China) on a Roche LightCycler 480 platform, where mRNA expression levels were normalised to β‐actin. All primers (Table 1) were commercially synthesised by Sangon Biotech (Shanghai, China).

**TABLE 1:** qPCR primer sequences.

Gene	Primers
*Il6*	F: TGATGGATGCTACCAAACTGGA
	R: TGACTCCAGCTTATCTCTTGGT
*Il18*	F: GTGAACCCCAGACCAGACTG
	R: CCTGGAACACGTTTCTGAAAGA
*Mcp1*	F: TGACCCCAAGAAGGAATGGG
	R: ACCTTAGGGCAGATGCAGTT
*Tnfa*	F: CCCTCACACTCACAAACCAC
	R: ACAAGGTACAACCCATCGGC
*Isg15*	F: TGGTACAGAACTGCAGCGAG
	R: AGCCAGAACTGGTCTTCGTG

### 2.12 Western blot

Total proteins were isolated from kidney tissue or the cell pellet with RIPA buffer (Beyotime). Proteins were electrophoresed by SDS‐PAGE, transferred to PVDF membranes, blocked with 5% skim milk (90 min, room temperature) and incubated with primary antibodies overnight at 4°C: anti‐NLRP3 (1:1000; ZEN‐BIOSCIENCE, China), anti‐CASP1 (1:1000; BOSTER, China), anti‐GSDMD (1:1000; HuaBio), anti‐Cleaved caspase‐1 (1:1000; Cell Signaling Technology, USA), anti‐STING (1:1000; Proteintech, USA), anti‐cGAS (1:1000; Abclone, China), anti‐TBK1 (1:1000, Proteintech), anti‐p‐TBK1 (1:1000; Proteintech), anti‐p65 (1:1000; absin, China), anti‐p‐p65 (1:1000; absin), anti‐ISG15 (1:1000; BOSTER). After being washed with PBST, the PVDF membranes were incubated with goat anti‐rabbit IgG (1:10 000; HuaBio, China) at room temperature for 1 h and then detected with ECL (Beyotime).

### 2.13 Renal histopathology

Kidney samples were fixed in 4% paraformaldehyde, paraffin‐embedded and stained with PAS or MASSON for histopathological analysis.

### 2.14 RNA sequencing analysis

Renal cortical RNA was isolated from DKD model mice (WT and KO). RNA quality was assessed using an Agilent 2100 Bioanalyzer (Agilent Technologies, USA) and RNase‐free agarose gel electrophoresis. RNA sequencing was performed by Gene Denovo Biotechnology (Guangzhou, China) using Illumina Novaseq6000 following standard protocols.

### 2.15 Statistical analysis

Data were expressed as the mean ± standard deviation (SD). Statistical analysis was performed using one‐way ANOVA (multiple groups) or Student's *t*‐test (pairwise comparisons) in GraphPad Prism 9.0. Experiments included ≥3 biological replicates, with reproducibility detailed in figure legends. Significance was set at *p* < .05.

## 3 RESULTS

### 3.1 High ISG15 expression in DKD mice

**FIGURE 1:**
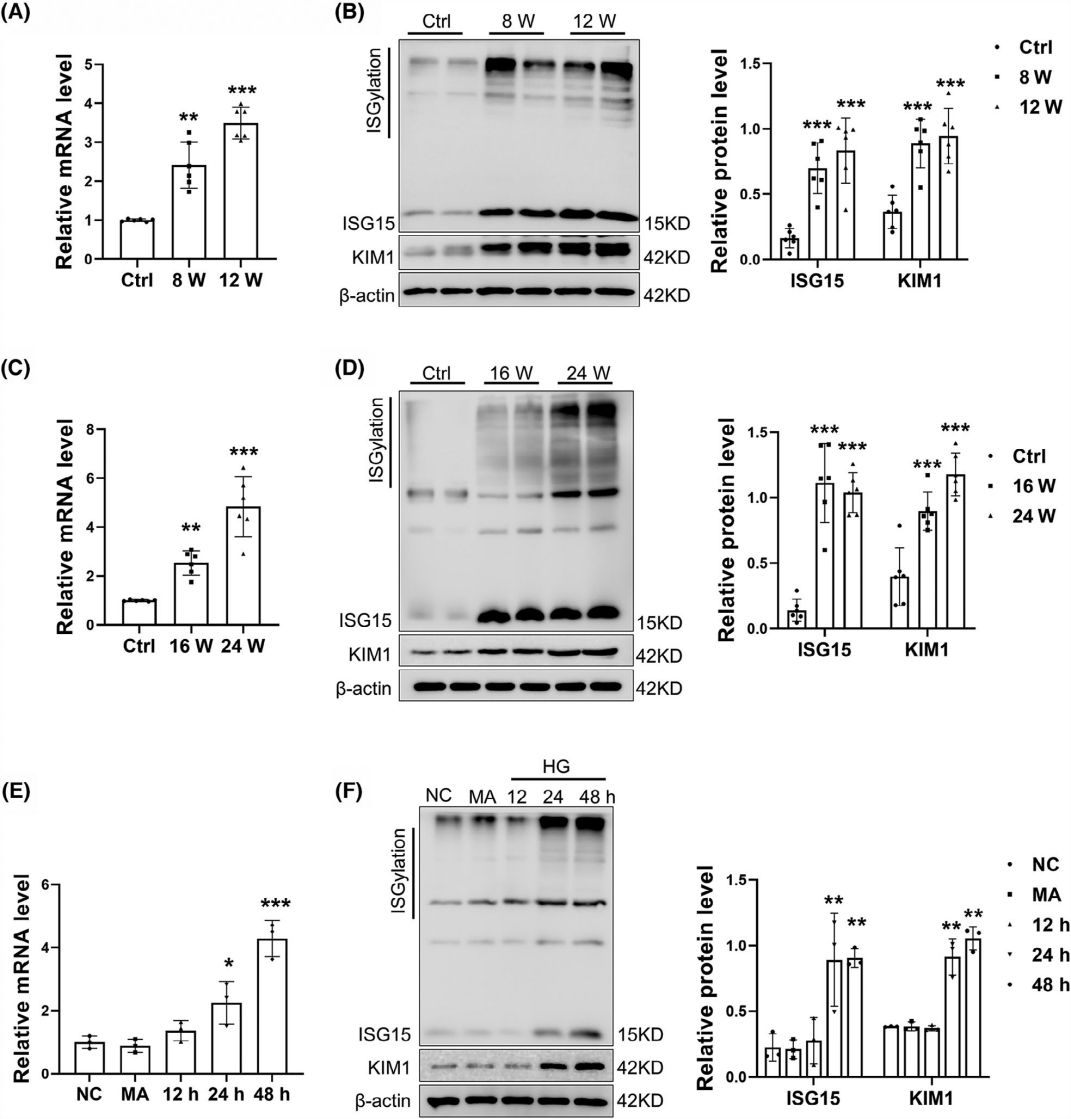
High ISG15 expression in DKD mice. (A) Relative mRNA level of *Isg15* in the kidney cortical tissues from WT mice and STZ/HFD‐induced DKD mice (*n* = 6). (B) Western blot analysis and quantification of ISG15/ISGylation and KIM‐1 expression in kidney cortical tissues from WT mice and STZ/HFD‐induced DKD mice (*n* = 6). (C) Relative mRNA level of *Isg15* in the kidney from db/m, 16 W db/db and 24 W db/db mice (*n* = 6). (D) Western blot analysis and quantification of ISG15/ISGylation and KIM‐1 expression in kidney cortical tissues from db/m, 16 W db/db and 24 W db/db mice (*n* = 6). (E) Relative mRNA level of *Isg15* in the TECs cultured in normal medium, MA or HG for 48 h (*n* = 3). (F) Western blot analysis and quantification of ISG15/ISGylation and KIM‐1 expression in the TECs (*n* = 3). DKD, diabetic kidney disease; HFD, high‐fat diet; HG, high glucose; STZ, streptozotocin; MA, mannitol. Results are expressed as the mean ± SD. *p < .05; **p < .01; ***p < .001.

To assess the relevance of ISG15 to DKD, we first examined ISG15 expression. By mRNA and Western blot analyses, we found elevated levels of *Isg15*, monomeric ISG15 (monISG15) protein expression and protein ISGylation in the kidney of STZ/HFD‐induced DKD mice. This increase correlated with elevated KIM1 expression, a tubular injury marker (Figure 1A,B). Similar results were obtained from db/db mice (Figure 1C,D), a model of spontaneous type 2 diabetes. Furthermore, an up‐regulation of ISG15, both at the mRNA and protein levels in TECs, was observed following treatment with HG, with the optimal time being determined at 48 h post‐treatment (Figures 1E,F and S1A–D). To ascertain whether the changes in ISG15 expression were due to high osmotic pressure, which can accompany HG exposure, TECs were treated with MA, a reagent used to mimic osmotic stress. Our findings revealed that MA‐induced osmotic pressure did not alter the expression of ISG15, indicating that the up‐regulation of ISG15 in TECs is predominantly triggered by HG rather than changes in osmotic pressure. In addition, increased expression of KIM1 and protein ISGylation was also observed in HG‐treated TECs. Collectively, these findings indicate an increase in ISG15 expression within the renal tubules of diabetic kidney tissues. This up‐regulation implies that ISG15 may serve as a novel mediator in the development of DKD, opening avenues for further exploration into its mechanistic contributions to DKD.

### 3.2 ISG15 deletion was protective against renal injury

**FIGURE 2:**
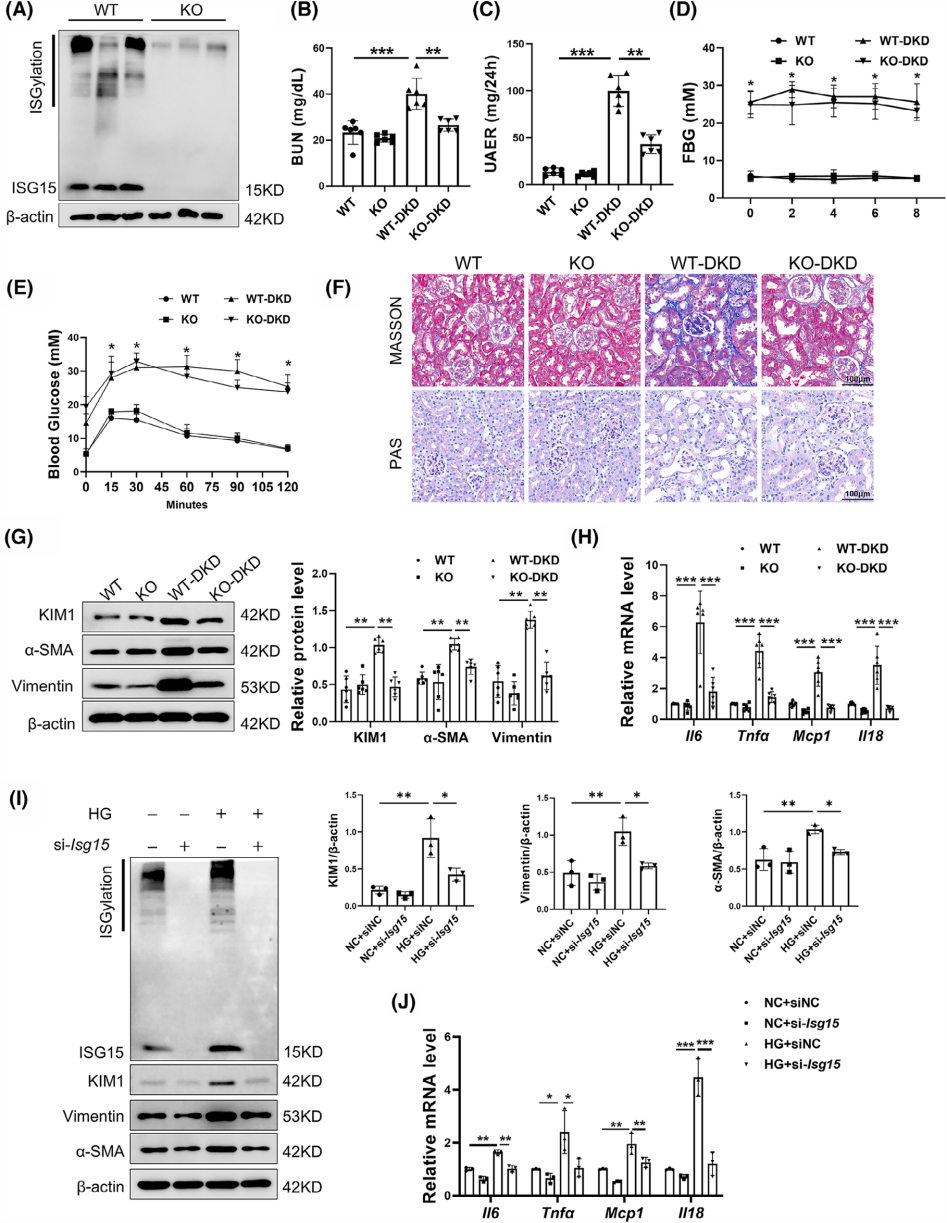
ISG15 deletion was protective against renal injury. (A) Western blot analysis and densitometric quantification of ISG15 expression in kidney tissues from WT and KO mice (*n* = 6). (B–E) BUN (B), UAER (C), FBG (D) and OGTT (E) levels in WT and KO mice treated with vehicle or STZ (*n* = 6). (F) Representative images of MASSON and PAS staining of the kidney (*n* = 6). (G) Western blot analysis and densitometric quantification of KIM1, α‐SMA and Vimentin expression in kidney tissues from WT and KO mice treated with vehicle or STZ (*n* = 6). (H) Relative mRNA level of pro‐inflammatory factors (*Il6*, *Tnfa, Mcp1* and *Il18*) in the kidney tissues from WT and KO mice treated with vehicle or STZ (*n* = 6). (I) Western blot analysis and densitometric quantification of ISG15/ISGylation, KIM1, α‐SMA and Vimentin expression in TECs (*n* = 3). (J) Relative mRNA level of pro‐inflammatory factors (*Il6, Tnfa, Mcp1* and *Il18*) in TECs (*n* = 3). TECs were transfected with sinc (50 nM) or si‐*Isg15* (50 nM), and then cultured in HG medium for 48 h. BUN, blood urea nitrogen; UAER, urinary albumin excretion rates; FBG, fasted blood; OGTT, oral glucose tolerance. Results are expressed as the mean ± SD. *p < .05; **p < .01; ***p < .001.

To further evaluate the role of ISG15 in DKD progression, a mouse model with global knockout of ISG15 was established and validated by Western blot (Figure 2A). Biochemical results demonstrated that there was no significant disparity between KO mice and WT mice. However, in these diabetic KO‐DKD mice compared with their WT‐DKD counterparts, we observed a reduction in blood urea nitrogen (BUN) and urinary albumin excretion rates (UAER), despite no discernible change in oral glucose tolerance test (OGTT) and fasted blood glucose (FBG) (Figure 2B–E). Additionally, there were no significant differences in PAS staining and Masson staining between KO mice and WT mice, indicating that the renal tissue structures of both types of mice were relatively normal and showed no obvious fibrosis. Moreover, ISG15 deficiency was found to significantly mitigate STZ/HFD‐induced renal tubular injury, as demonstrated by MASSON and PAS staining (Figure 2F). MASSON staining showed elevated in profibrotic collagen deposition in DKD mice. However, the knockout of ISG15 led to a marked reduction in collagen deposition, effectively mitigating renal fibrosis. PAS staining revealed a large amount of glycogen deposition in the renal cortex of DKD mice, which is associated with disruptions in the structure of the renal tubules and significant tubular dilation. In contrast, the specific genetic ablation of ISG15 leads to a marked improvement in the structural integrity of the kidneys and a noticeable reduction in tubular dilation. The abnormal expression of fibrosis proteins and inflammatory cytokines within the renal is a pivotal factor in the progression of DKD.^22^, ^23^ In line with this, we assessed tubular cells fibrosis and inflammation. Notably, the expression of KIM1 and fibrotic markers (Vimentin and α‐SMA) was significantly decreased in ISG15‐deficient mice after STZ/HFD treatment, while no significant differences were detected between untreated WT and KO mice (Figure 2G). Furthermore, the inflammatory response was attenuated, as evidenced by decreased levels of *Mcp‐1*, *Il‐18*, *Il‐6* and *Tnf‐α* (Figure 2H). In summary, knockout of ISG15 did not alter the renal physiology and biochemistry in mice. However, compared with the model group, KO‐DKD mice exhibited a delayed onset of fibrosis and inflammation. Together, these findings indicated that the up‐regulation of ISG15 in mice increases their susceptibility to renal tubular dysfunction and DKD development under diabetic conditions, suggesting that ISG15 may as a potential therapeutic target in DKD management.

In order to further confirm the role of ISG15 in DKD, ISG15 was knockdown via transfecting siRNA against *Isg15* into the TECs. Consistent with in vivo results, si‐*Isg15* significantly decreased ISG15/ISGylation levels and attenuated HG‐induced fibrotic and inflammatory responses in TECs compared with siNC (Figure 2I,J). Taken together, these results further demonstrated that ISG15 promotes tubular cell injury, thereby accelerating the development of DKD.

### 3.3 Ablation of ISG15 decreased pyroptosis of TECs under HG stimulation

**FIGURE 3:**
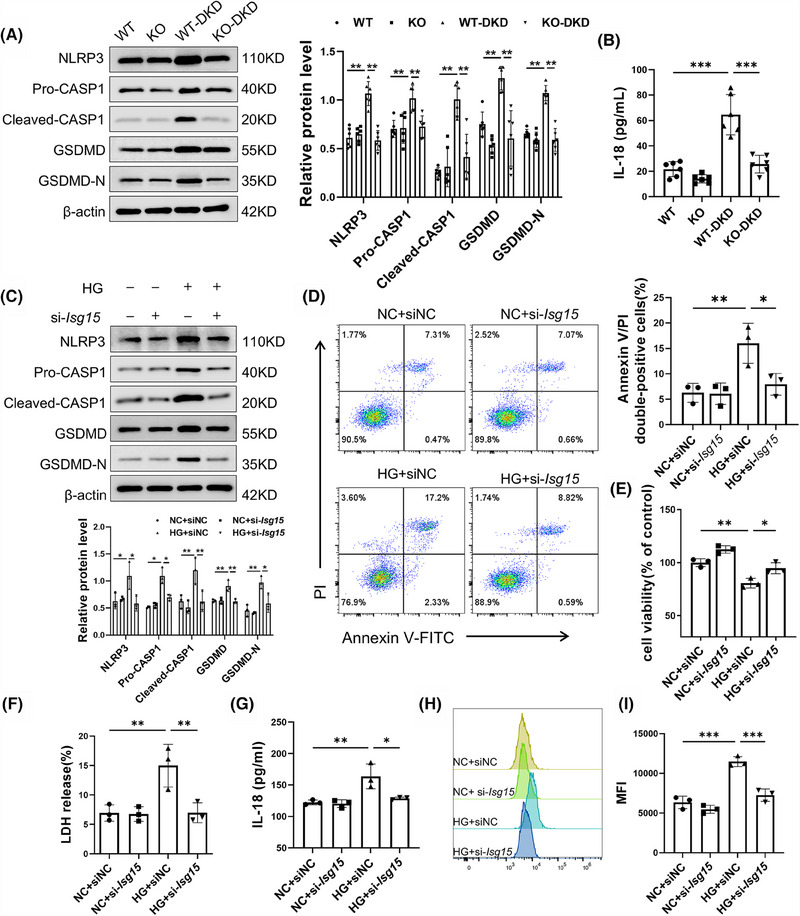
Ablation of ISG15 decreased pyroptosis of TECs under HG stimulation. (A) Western blot analysis and densitometric quantification of pyroptosis‐related proteins (NLRP3, Pro‐CASP1, Cleaved‐CASP1, GSDMD, GSDMD‐N) expression in kidney tissues from WT and KO mice treated with vehicle or STZ (*n* = 6). (B) Level of IL‐18 in the serum from WT and KO mice treated with vehicle or STZ (*n* = 6). (C) Western blot analysis of pyroptosis‐related proteins (NLRP3, Pro‐CASP1, Cleaved‐CASP1, GSDMD, GSDMD‐N) expression in TECs (*n* = 3). (D) Flow cytometry analysis and quantitative data depicting the TECs Annexin V/PI double‐positive cells rate (*n* = 3). (E) CCK‐8‐kit activity assay quantified cell viability (*n* = 3). (F–I) Level of LDH (F), IL‐18 (G), ROS (H and I) in TECs (*n* = 3). TECs were transfected with sinc (50 nM) or si‐*Isg15* (50 nM), and then cultured in HG medium for 48 h. Results are expressed as the mean ± SD. *p < .05; **p < .01; ***p < .001.

To elucidate the role of ISG15 in the downstream effects contributing to TECs fibrosis, inflammation and renal failure, we have zeroed in on the significance of pyroptosis, a vital innate immune response. Pyroptosis is known to trigger a cascade of events: the release of cytokines, a robust inflammatory response, and ultimately, the activation of immune phagocytosis.^24^ Notably, the expression of key pyroptosis‐associated proteins was all found to be up‐regulated in DKD (Figure S2A). Our experiments using AAV9 to knockdown GSDMD in DKD mice significantly alleviated renal fibrosis and inflammation (Figure S2B,C), underscoring the pivotal role of pyroptosis in these pathological processes. Given the elevated expression level of ISG15 observed in DKD, we proceeded to investigate its potential relationship with pyroptosis. The results, as depicted in Figure 3A, reveal that ISG15 deficiency notably mitigated pyroptosis in renal tissues compared with DKD mice, evidenced by the decreased expression of NLRP3, CASP1 and GSDMD. Furthermore, the level of IL‐18 in the serum, an indicator of pyroptosis activity, was also diminished in ISG15‐deficiency mice as determined by ELISA assays (Figure 3B). Meanwhile, there was no significant difference between WT and KO mice. In vitro studies further substantiated the role of ISG15 in pyroptosis. Western blot analysis revealed that ISG15 silencing markedly reduced the up‐regulation of pyroptosis‐associated proteins triggered by high glucose conditions (Figure 3C). Moreover, ISG15 knockdown reduced the rate of Annexin V/PI double‐positive cells under HG conditions (Figure 3D), whereas a concomitant increase in cell viability was observed through the CCK‐8 assay (Figure 3E). Additionally, transfection of the si‐*Isg15* markedly decreased the release rate of LDH and IL‐18, both indicative of pyroptosis (Figure 3F,G), and the level of ROS (Figure 3H,I), reinforcing the central role of ISG15 in mediating the pyroptosis of TECs.

### 3.4 Inhibition of pyroptosis blocked TECs damage and fibrosis induced by ISG15

**FIGURE 4:**
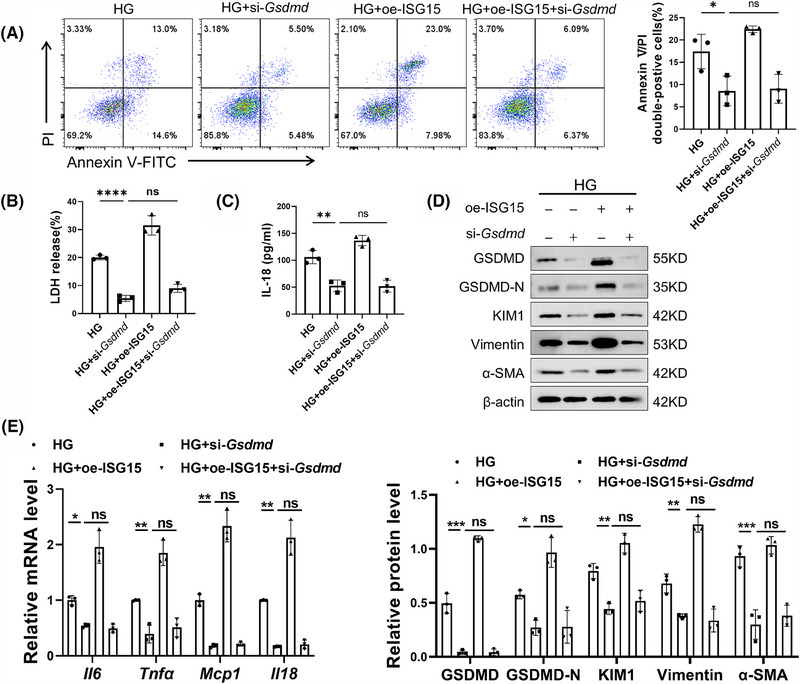
Inhibition of pyroptosis blocked TECs damage and fibrosis induced by ISG15. (A) Flow cytometry analysis and quantitative data depicting the Annexin V/PI double‐positive cells rate (*n* = 3). (B and C) Levels of LDH (B), IL‐18 (C) in TECs (*n* = 3). (D) Western blot analysis and densitometric quantification of GSDMD, GSDMD‐N, KIM1, α‐SMA and Vimentin expression in TECs (*n* = 3). (E) Relative mRNA level of pro‐inflammatory factors (*Il6*, *Tnfa*, *Mcp1* and *Il18*) in TECs (*n* = 3). TECs were transfected with si‐*Gsdmd* (50 nM) or oe‐ISG15 (4 µg), and then cultured in HG medium for 48 h. Results are expressed as the mean ± SD. *p < .05; **p < .01; ***p < .001.

To elucidate the necessity of pyroptosis signalling in the ISG15‐induced promotion of DKD, we transferred specific siRNA into TECs to down‐regulate the expression of GSDMD, a key mediator of pyroptosis. As expected, knockdown of GSDMD reversed the ISG15‐mediated up‐regulation of Annexin V/PI double‐positive cells rate and the production of LDH and IL‐18 in HG‐induced TECs (Figure 4A–C). Western blot analysis showed that the expression of fibrotic markers such as Vimentin, α‐SMA and KIM1 was significantly decreased, further substantiated the role of GSDMD in the ISG15‐mediated TECs damage and fibrosis (Figure 4D). Additionally, GSDMD knockdown nearly completely suppressed ISG15‐mediated up‐regulation of inflammatory cytokines in TECs (Figure 4E).

Paralleling our approach with GSDMD‐deficient TECs, we also investigated the impact of NLRP3 inhibition on these processes. Pretreatment of TECs with the NLRP3 inhibitor MCC950 resulted in the suppression of NLRP3 signalling pathways (Figure S3A). Similar to the above findings in GSDMD‐deficient TECs, blockade of NLRP3 activation reversed the ISG15‐induced increase in the Annexin V/PI double‐positive cells rate and secretion of LDH and IL‐18 (Figure S3B–D). Furthermore, MCC950 treatment impaired the ISG15‐induced pro‐fibrosis effects and the production of inflammatory cytokines (Figure S3E,F). In summary, our findings collectively indicated that ISG15 exacerbates TECs pyroptosis via NLRP3, thereby contributing to tubular cell damage and fibrosis in DKD. These results underscore the potential therapeutic value of targeting ISG15 or its associated signalling pathways in the treatment of DKD.

### 3.5 ISG15 mediated HG‐induced mitochondrial impairment and mtDNA release

To assess ISG15's function in TEC pyroptosis, RNA‐seq analysis was performed on kidneys obtained from ISG15‐KO and WT mice with DKD. Our comparative analysis revealed 1593 differentially expressed genes, with 428 up‐regulated and 1165 down‐regulated in the ISG15‐KO mice (FDR < 0.05, |log2FC| > 1, *n* = 3; Figure S4A). The KEGG analysis of the down‐regulated genes highlighted their involvement in cell adhesion molecules, cytokine–cytokine receptor interactions, chemokine signalling pathway and NF‐κB signalling pathway (Figure S4B), indicating that ISG15 deficiency may attenuate fibrosis and inflammation in DKD. Interestingly, GSEA demonstrated significant down‐regulation of the NOD‐like receptor signalling pathway, which further validated our experimental results (Figure S4C).

**FIGURE 5:**
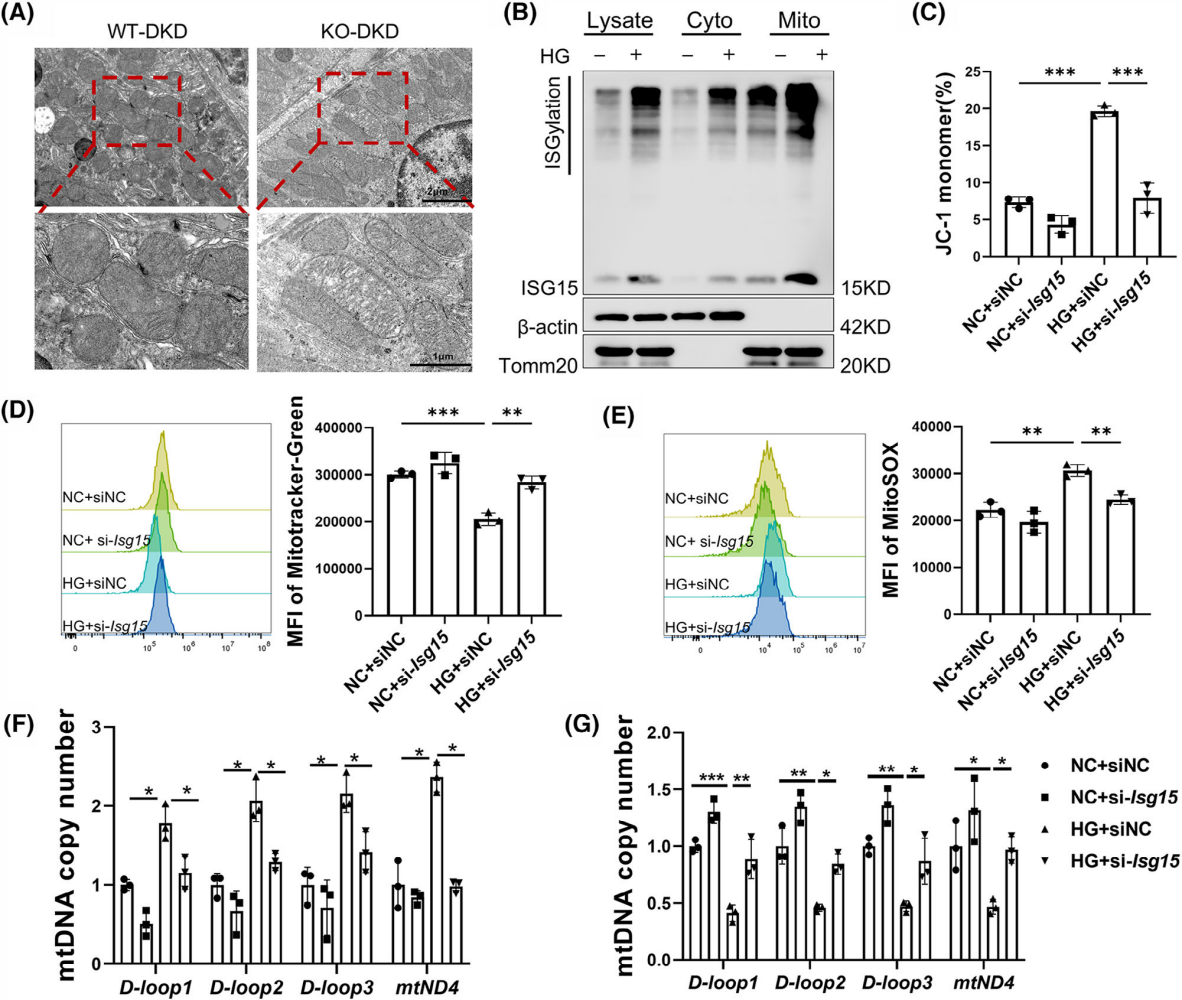
ISG15 was involved in HG‐induced mitochondrial impairment and mtDNA release. (A) Representative TEM images of kidney tissues from WT and KO mice treated with STZ (*n* = 6). (B) Western blot analysis ISG15/ISGylation expression in TECs treated with vehicle or HG (*n* = 3). (C–E) Flow cytometry analysis and quantitative data depicting the mitochondrial membrane potential (C), mitochondrial mass (D) and mtROS (E) (*n* = 3). (F and G) qPCR analysis the mtDNA (*Loop1‐3* and *mt‐Nd4*) copy number in the cytosolic compartments (F) and mitochondria (G) (*n* = 3). TECs were treated with vehicle or C‐176 (10 µM), and then cultured in HG medium for 48 h. Results are expressed as the mean ± SD. *p < .05; **p < .01; ***p < .001; ns, not significant.

Given the association between persistent HG stimulation and aberrant mitochondrial metabolism, which leads to elevated ROS levels in proximal renal tubular cells and contributes to DKD, we hypothesised a connection between ISG15 and mitochondrial function.^25^ This hypothesis was supported by previous associations of ISG15 with mitochondria and the high mitochondrial content in TECs.^26^, ^27^ In addition, the GSEA results also highlighted enrichment in the mitochondrial matrix, mitochondrial translation and mitochondrial gene expression within the KEGG pathways (Figure S4D). TEM imaging revealed that DKD mice exhibited exacerbated mitochondrial deformation, characterised by disintegrated cristae and vacuolisation, whereas deletion of ISG15 mitigated the abnormal mitochondrial morphology (Figure 5A). Additionally, the endogenous mono‐ISG15 and ISGylation localised to mitochondria was observed upon HG treatment, and overexpression of ISG15 in TECs also induced similar mitochondrial localisation (Figures 5B and S4E). These results imply that ISG15 may be involved in TECs pyroptosis by regulating mitochondrial homeostasis. To explore this hypothesis, we examined the mitochondrial membrane potential, mitochondrial mass and mtROS levels under HG stress with or without ISG15 silencing. JC‐1 analysis showed that HG‐induced loss of mitochondrial membrane potential was reversed by si‐*Isg15* treatment (Figure 5C). Similarly, the HG‐induced reduction in mitochondrial mass, as measured by Mito‐Tracker Green, was nullified by si‐*Isg15* (Figure 5D). Notably, as shown in Figure 5E, HG injury was found to stimulate TECs to produce excessive mtROS, as demonstrated via MitoSOX staining, a phenomenon that was attenuated by ISG15 knockdown. Oxidative stress is a vital factor in the release of mtDNA into the cytosol.^28^ Therefore, we quantified the mtDNA by isolating mitochondria and cytoplasm from the entire cell and performing qPCR analysis. As shown in Figure 5F, the enrichment of mtDNA in the cytosolic compartments, such as *Loop1‐3* and *mt‐Nd4*, was markedly elevated in HG‐treated cells but was reduced by ISG15 knockdown. Furthermore, ISG15 silencing attenuated HG‐induced mitochondrial mtDNA copy number reduction (Figure 5G). These findings indicated that ISG15 up‐regulation contributed to HG‐induced mitochondrial dysfunction and mtDNA release.

To ascertain the necessity of mtROS in ISG15‐mediated leakage, we co‐treated ISG15‐overexpression cells with mitoTEMPO, a specific scavenger of mtROS. Results showed that mtROS overproduction, induced by ISG15 overexpression, was alleviated by mitoTEMPO (Figure S5A). ISG15 overexpression elevated NLRP3, GSDMD, GSDMD‐N and KIM1 expression, as shown by Western blot, with mitoTEMPO reversing these effects (Figure S5B). These integrated results suggested that mtROS plays a crucial role in ISG15‐induced TECs injury.

### 3.6 The cGAS–STING pathway was activated in the DKD mice

**FIGURE 6:**
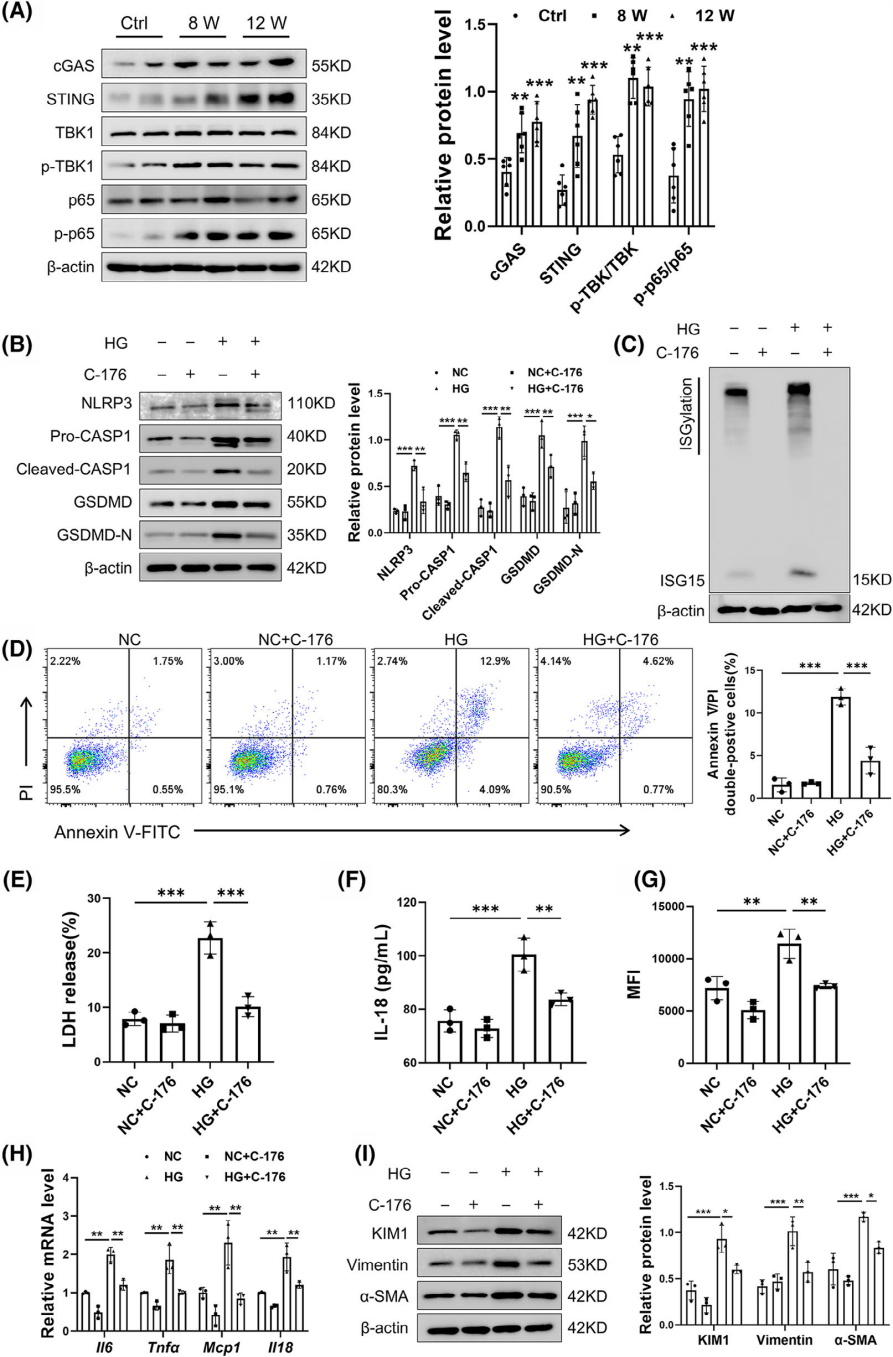
The cGAS–STING pathway was activated in the DKD mice. (A) Western blot analysis and densitometric quantification of cGAS, STING, TBK1, p‐TBK1, p65, p‐p65 expression in DKD mice (*n* = 6). (B) Western blot analysis and densitometric quantification of NLRP3, Pro‐CASP1, Cleaved‐CASP1, GSDMD, GSDMD‐N expression in TECs (*n* = 3). (C) Western blot analysis ISG15/ISGylation expression in TECs (*n* = 3). (D) Flow cytometry analysis and quantitative data depicting the Annexin V/PI double‐positive cells rate (*n* = 3). (E–G) Levels of LDH (E), IL‐18 (F), ROS (G) in TECs (*n* = 3). (H) Relative mRNA level of pro‐inflammatory factors (*Il6*, *Tnfa*, *Mcp1*, *Il18*) in TECs (*n* = 3). (I) Western blot analysis and densitometric quantification of KIM1, α‐SMA and Vimentin expression in TECs (*n* = 3). TECs were transfected with vehicle or C‐176 (10 µM), and then cultured in HG medium for 48 h. Results are expressed as the mean ± SD. *p < .05; **p < .01; ***p < .001.

Given that cGAS is recognised as a cytoplasmic DNA biosensor, the cGAS–STING pathway was investigated for its activation in DKD mice. Western blot analysis revealed heightened activation of the cGAS–STING pathway in DKD mice, as indicated by increased protein levels of cGAS, STING, p‐p65/p65 and p‐TBK1/TBK1 (Figure 6A). To ascertain the necessity of the cGAS–STING pathway in TECs damage, C‐176, a pharmacological inhibitor of STING, was added to HG‐treated TECs. Western blot results showed that C‐176 reversed the HG‐induced up‐regulation of pyroptosis‐associated core proteins (Figure 6B). Intriguingly, previous research has indicated that STING is capable of inducing the expression of ISG15.^29^, ^30^, ^31^ In a striking parallel, our findings reveal that the inhibition of STING by C‐176 correspondingly suppressed the expression of ISG15 (Figure 6C). Further investigation showed that C‐176 mitigated the injurious effects of HG on TECs, as indicated by a decrease in Annexin V/PI double‐positive cells rate (Figure 6D). Additionally, the secretion of LDH and IL‐18 in TECs culture supernatants, as well as the levels of ROS, were decreased following STING inhibition (Figure 6E–G). The proinflammatory cytokines induced by HG in TECs were also mitigated by C‐176 (Figure 6H). Moreover, Inhibition of STING in TECs decreased KIM1 and fibrosis markers (Vimentin and α‐SMA), underscoring its contribution to fibrotic processes (Figure 6I). In summary, these findings suggested that the cGAS–STING pathway modulates ISG15 expression and contributes to HG‐induced TECs damage, highlighting its potential as a DKD therapeutic target.

### 3.7 ISG15 promoted STING signalling via cytosolic mtDNA and established a positive feedback loop

**FIGURE 7:**
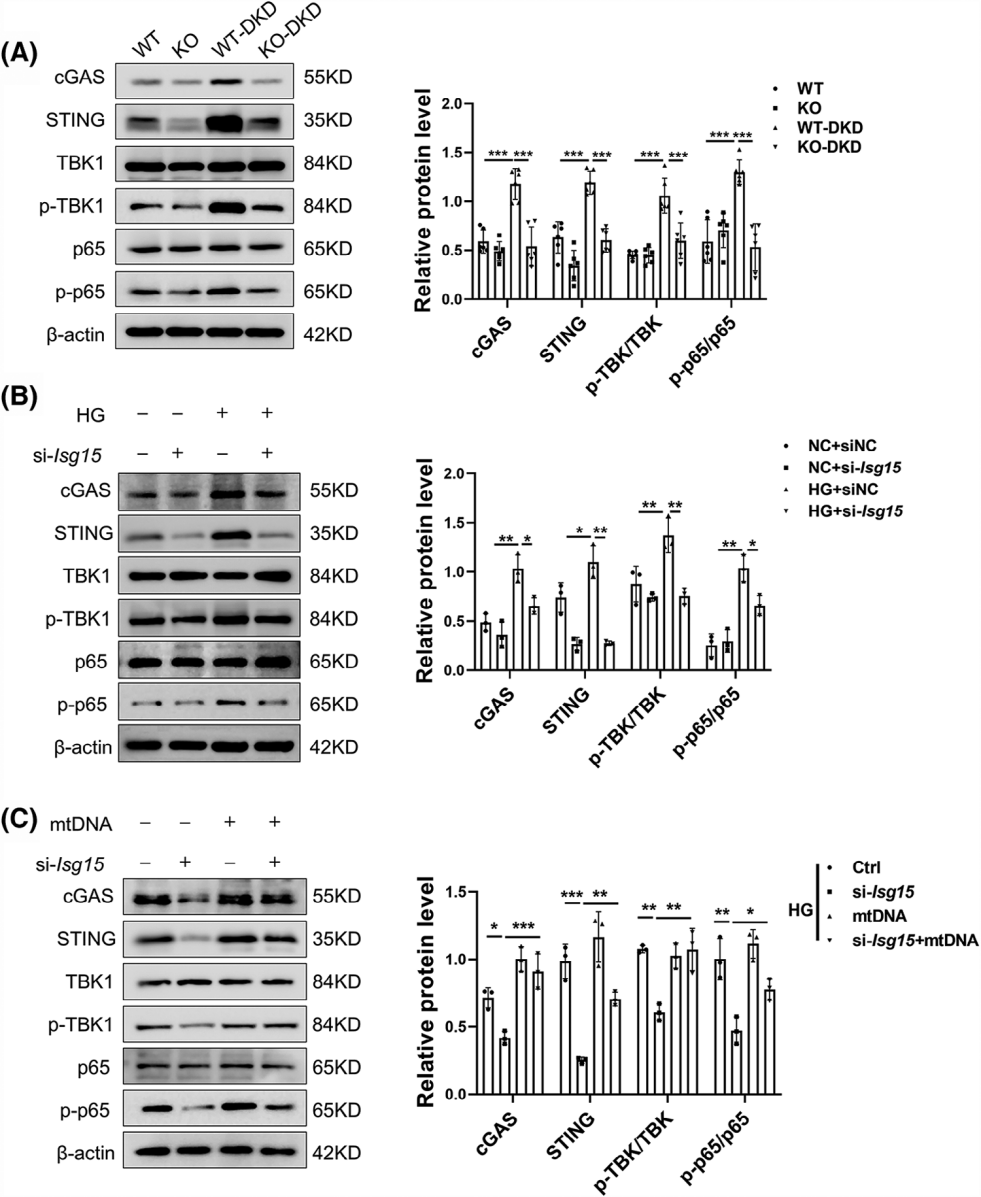
ISG15 promoted STING signalling via cytosolic mtDNA and established a positive feedback loop. (A) Western blot analysis and densitometric quantification of cGAS, STING, TBK1, p‐TBK1, p65, p‐p65 expression in WT and KO mice treated with vehicle or STZ (*n* = 6). (B and C) Western blot analysis and densitometric quantification of cGAS, STING, TBK1, p‐TBK1, p65, p‐p65 expression in TECs (*n* = 3). TECs were transfected with mtDNA (4 µg) or si‐*Isg15* (50 nM), and then cultured in HG medium for 48 h. Results are expressed as the mean ± SD. *p < .05; **p < .01; ***p < .001.

To decipher ISG15‐initiated signalling, we explored the interplay among ISG15, cytosolic mtDNA and the cGAS–STING pathway. Our study demonstrated that ISG15 deletion significantly inhibited the activation of cGAS–STING pathway when compared with STZ/HFD controls (Figure 7A). This was further corroborated in HG‐stimulated TECs with si‐*Isg15*, as evidenced by the down‐regulation of pathway proteins, such as cGAS, STING, p‐p65/p65 and p‐TBK1/TBK1 (Figure 7B). These results suggested that ISG15 is a pivotal regulator of gene expression related to the STING pathway. Considering the association between cytosolic mtDNA and the STING pathway, we examined the relationship between ISG15 expression and the mtDNA‐dependent STING pathway. Upon stimulation with mtDNA, the STING pathway in HG‐treated TECs was activated, and this activation was mitigated by si‐*Isg15* treatment (Figure 7C). Our findings revealed that the TECs‐intrinsic ISG15 activation enhanced mtDNA‐mediated STING pathway activation, thereby establishing a positive feedback mechanism.

### 3.8 ISG15–STING loop‐maintained HG‐induced injury in TECs

**FIGURE 8:**
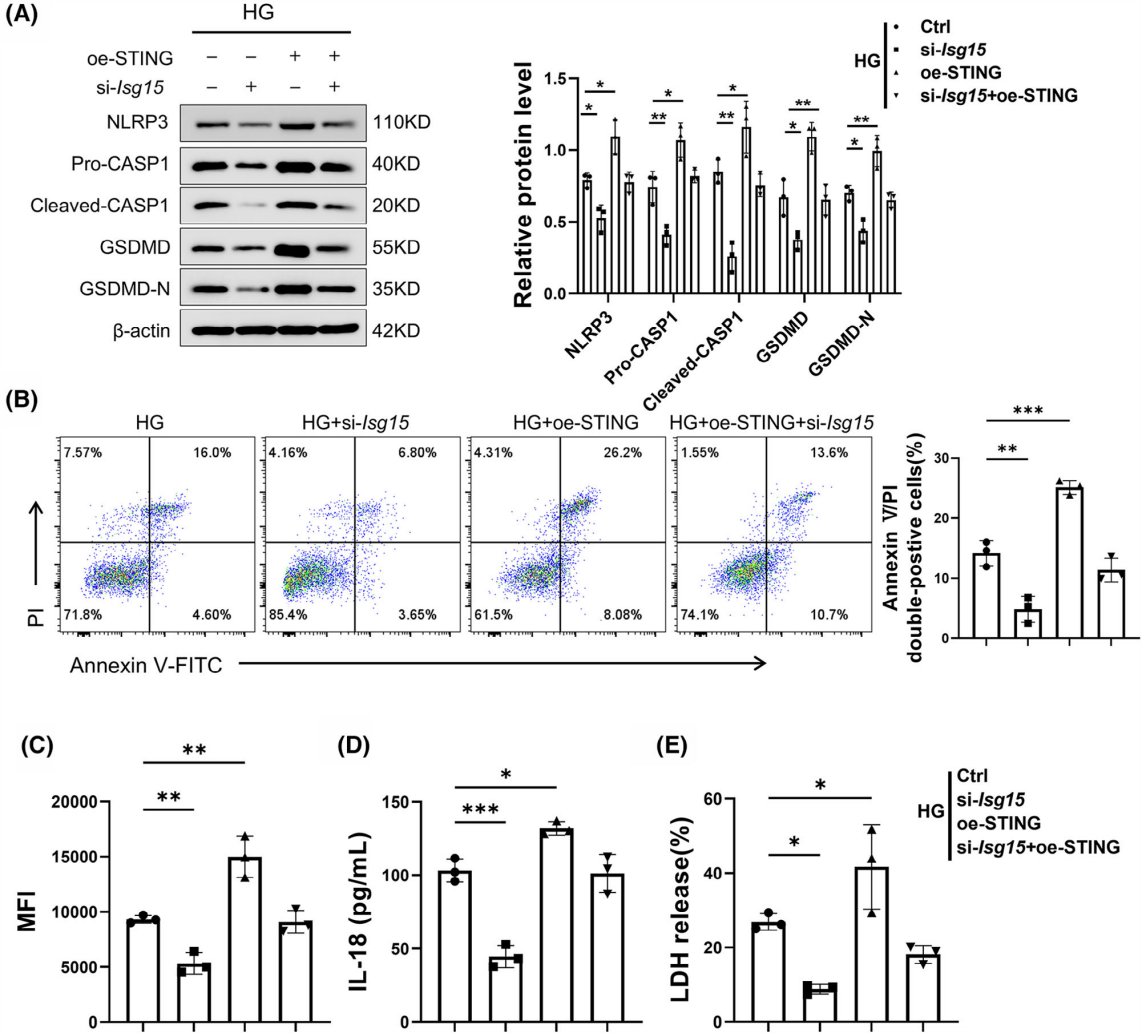
ISG15–STING loop‐maintained HG‐induced injury in TECs. (A) Western blot analysis and densitometric quantification of NLRP3, Pro‐CASP1, Cleaved‐CASP1, GSDMD and GSDMD‐N expression in TECs (*n* = 3). (B) Flow cytometry analysis and quantitative data depicting the Annexin V/PI double‐positive cells rate (*n* = 3). (C–E) Levels of ROS (C), IL‐18 (D), LDH (E) in TECs (*n* = 3). TECs were transfected with oe‐STING (4 µg) or si‐*Isg15* (50 nM), and then cultured in HG medium for 48 h. Results are expressed as the mean ± SD. *p < .05; **p < .01; ***p < .001.

We hypothesised that the elevated STING‐dependent pyroptosis phenotype is pivotal in the development of renal failure and fibrosis induced by ISG15. In order to verify our assumption, si‐*Isg15* was transfected into TECs with or without STING overexpression vector. In line with our assumption, STING overexpression neutralised si‐*Isg15*‐mediated pyroptosis suppression in TECs, with pyroptosis marker levels returning to baseline (Figure 8A). Additionally, the reduction in Annexin V/PI double‐positive cells rate induced by si‐*Isg15* was partially reversed by STING overexpression in HG‐induced TECs (Figure 8B). Further investigation revealed that STING overexpression also counteracted the effects of ISG15 knockdown mediated on the release of ROS, LDH and IL‐18 (Figure 8C–E). In addition, we assessed influence of STING on protein expression associated with fibrosis and TECs injury. Results showed that the down‐regulation of KIM1, Vimentin and α‐SMA proteins induced by si‐*Isg15* was markedly reversed by STING overexpression (Figure S6A). Moreover, the effect of si‐*Isg15* on the production of proinflammatory cytokines were diminished by STING overexpression (Figure S6B). Collectively, our results demonstrate that ISG15 silencing attenuates HG‐induced TECs injury through modulation of the STING pathway, suggesting its therapeutic potential for renal diseases.

In addition, we transfected an ISG15 overexpression vector into TECs treated with vehicle or C‐176. Pharmacological inhibition of STING with C‐176 effectively counteracted the oe‐ISG15‐driven elevation of pyroptosis‐associated proteins, fibrosis‐related proteins and KIM1 (Figure S7A,B). Furthermore, inhibition of STING impaired the overexpression of ISG15‐induced up‐regulation of inflammatory cytokines (Figure S7C). These data indicated that the ISG15–STING loop plays a crucial role in maintaining HG‐induced injury in TECs.

### 3.9 ISG15 contributed to TECs injury in an ISGylation‐dependent manner

**FIGURE 9:**
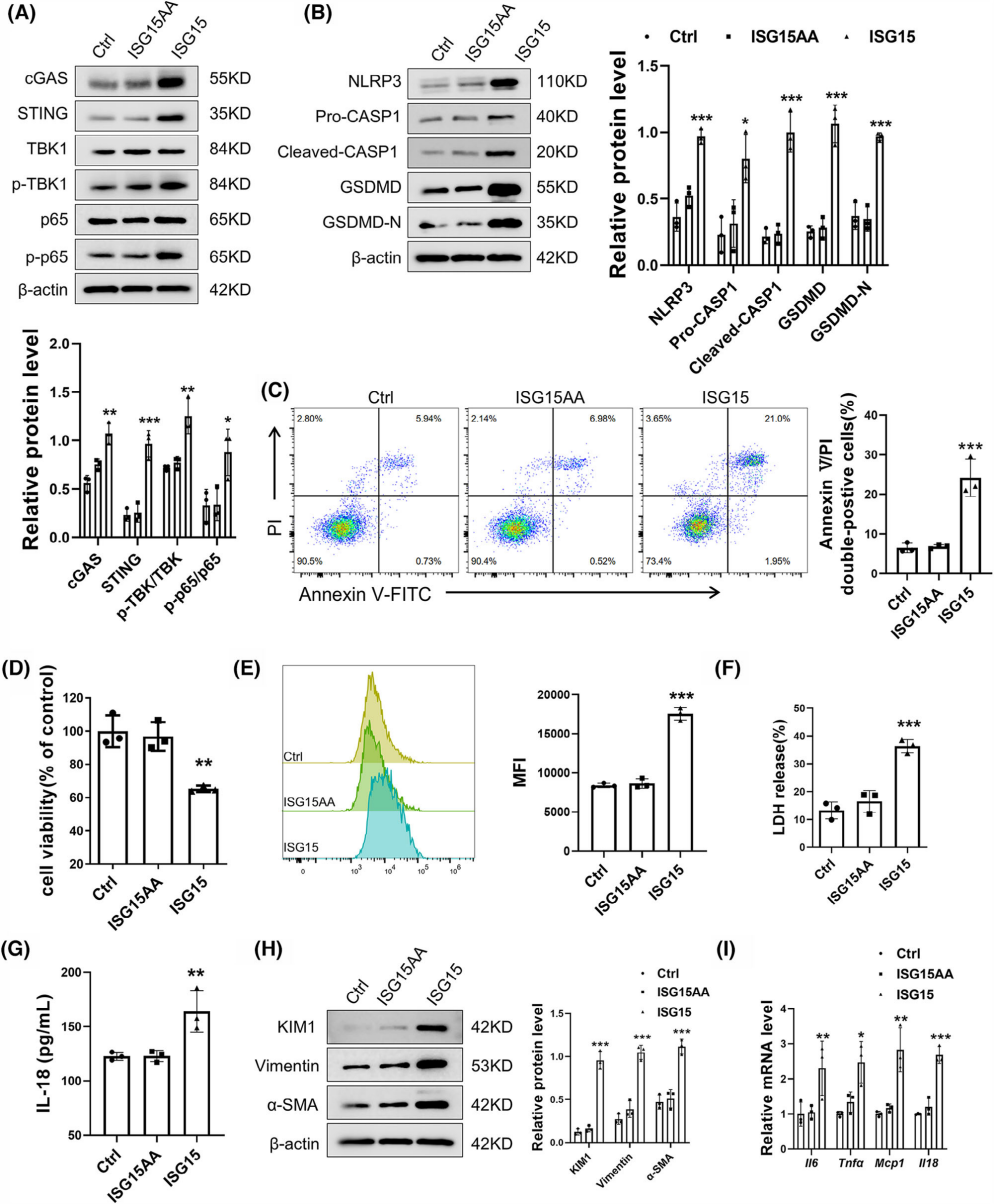
ISG15 contributed to TECs injury in an ISGylation‐dependent manner. (A) Western blot analysis and densitometric quantification of cGAS, STING, TBK1, p‐TBK1, p65, p‐p65 expression in TECs (*n* = 3). (B) Western blot analysis and densitometric quantification of NLRP3, Pro‐CASP1, Cleaved‐CASP1, GSDMD, GSDMD‐N expression in TECs (*n* = 3). (C) Flow cytometry analysis and quantitative data depicting the Annexin V/PI double‐positive cells rate (*n* = 3). (D) CCK‐8 activity assay quantified cell viability (*n* = 3). (E–G) Levels of ROS (E), LDH (F), IL‐18 (G) in TECs (*n* = 3). (H) Western blot analysis and densitometric quantification of KIM1, α‐SMA and Vimentin expression in TECs (*n* = 3). (I) Relative mRNA level of pro‐inflammatory factors (*Il6*, *Tnfa*, *Mcp1* and *Il18*) in TECs (*n* = 3). TECs were transfected with empty vector, ISG15AA or ISG15 (4 µg). Results are expressed as the mean ± SD. *p < .05; **p < .01; ***p < .001.

ISG15 is a versatile protein that exists as a free protein and as a conjugate to other proteins. The exposure of its C‐terminal LRLRGG motif is crucial for the conjugation of ISG15 to substrates.^32^, ^33^ To elucidate whether ISG15 exerts its effects in its free form or as a conjugate, TECs were transfected with three different vectors: an empty vector, an ISG15 (wild‐type) vector or an unconjugatable ISG15‐AA mutant vector. As shown in Figure 9A, cells with functional ISGylation machinery exhibited enhanced levels of key signalling components (cGAS, STING) and their downstream effectors. Notably, protein levels showed no significant differences between empty vector‐ and ISG15AA vector‐transfected cells. The results confirmed that the ISGylation activation rather than mono‐ISG15 is essential for the cGAS–STING pathway. Additionally, the up‐regulation of ISGylation, not the mere presence of ISG15, promoted the expression of pyroptosis‐associated proteins and Annexin V/PI double‐positive cell rate of TECs (Figure 9B,C). CCK‐8 analysis revealed that ISG15 overexpression decreased the cell viability compared with those expressing ISG15AA (Figure 9D). Moreover, the increase of ISGylation contributed to the release rate of ROS, LDH and IL‐18 (Figure 9E–G). Western blot and qPCR showed that ISGylation, not mono‐ISG15, exacerbated the activated fibrosis and inflammation in TECs (Figure 9H–I). The overexpression of USP18 in TECs, an ISG15‐specific protease that removes it from substrate proteins, effectively mitigates the deleterious effects induced by HG, further confirmed role of ISGylation (Figure S8A–D). In summary, these results demonstrated that ISG15 promoted TECs damage in an ISGylation‐dependent manner, rather than through its monomeric form.

## 4 DISCUSSION

**FIGURE 10:**
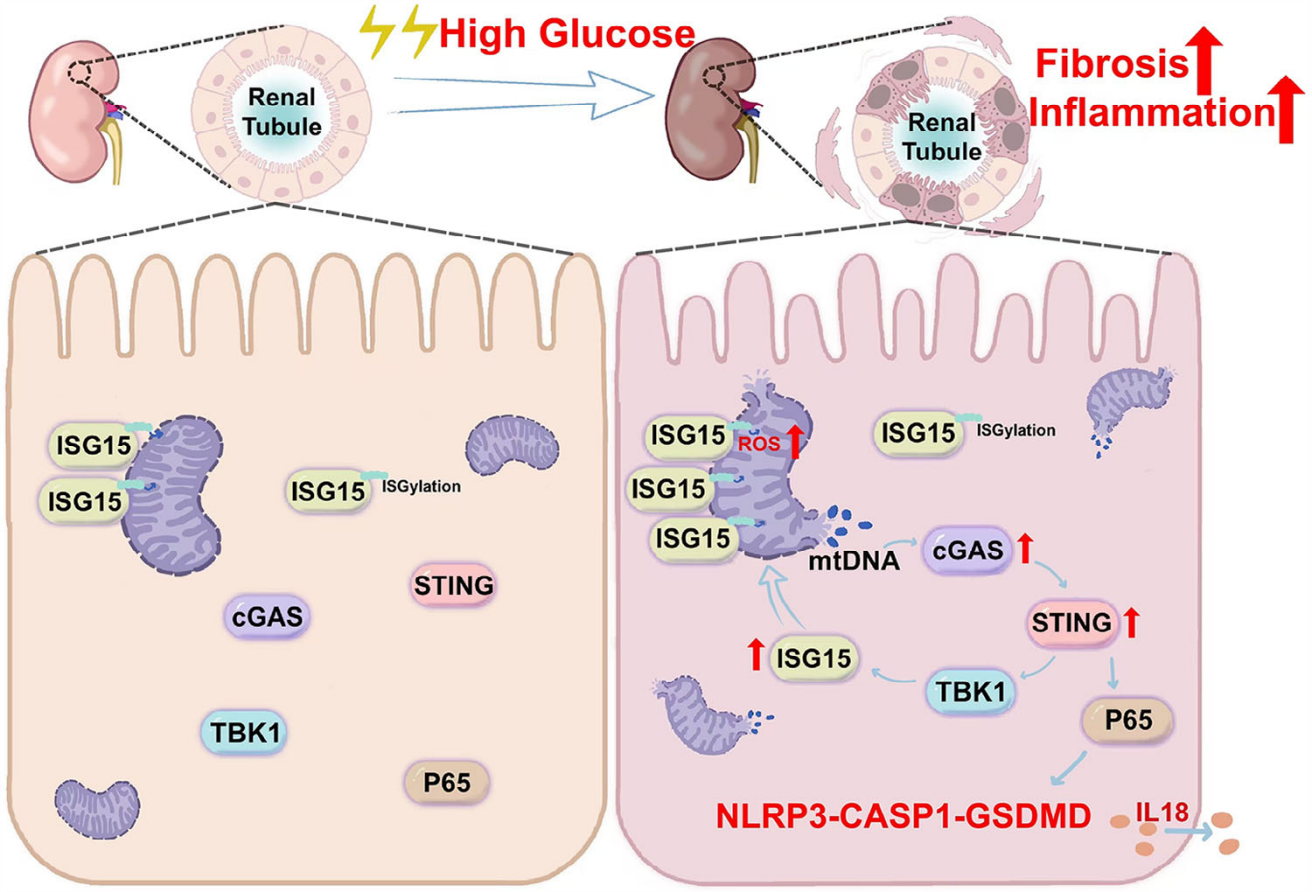
ISG15 up‐regulation in TECs aggravated DKD progression via mtDNA mediated cGAS–STING signalling. The up‐regulation of ISG15 in TECs under HG stimulation disrupted mitochondrial homeostasis and triggered the release of mtDNA into the cytosol, which in turn activated the NLRP3‐mediated pyroptosis in a cGAS–STING‐dependent manner, and promoted the progression of DKD.

DKD represents a critical microvascular complication and is the primary contributor to end‐stage kidney disease globally.^34^ Despite its prevalence, effective treatment options for DKD are lacking. Increasing evidence showed the involvement of fibrosis and inflammation in the progression of kidney damage.^35^, ^36^, ^37^ While initial studies on ISG15 mainly focused on its antiviral and immune modulation functions,^18^, ^38^ its specific role of ISG15 in the pathogenesis of DKD remains limited. In this work, we elucidated novel mechanisms underlying ISG15's role in DKD. We discovered an unusual up‐regulation of ISG15 at both transcriptional and translational levels. Also, ISG15 was found to disrupt mitochondrial homeostasis, triggering mtDNA release into the cytosol in TECs under HG conditions. This release of mtDNA activated the NLRP3‐mediated pyroptosis in a cGAS–STING‐dependent manner. Furthermore, ISGylation rather than mono‐ISG15, formed a regulatory loop with STING, which was closely related to TECs injury in DKD mice by modulating pyroptosis. These results clearly demonstrate that ISG15 up‐regulation acts as a catalyst for TECs fibrosis and inflammation, thereby promoting the development of DKD (Figure 10).

The development of DKD is driven by a multifaceted interplay of factors, including advanced glycation end product (AGE) accumulation, renin–angiotensin system activation, cellular stress, fibrotic processes and inflammatory responses. The better understanding of these interconnected pathophysiological mechanisms, particularly those involving haemodynamic, metabolic and inflammatory pathways, was helpful for the treatment of DKD.^39^ TECs, which constitute the predominant cellular component of renal parenchyma, play essential roles in kidney function maintenance. ISG15 is a multifunctional molecule known for its regulatory role in various signalling pathways by directly modulating the function of host and viral proteins. In this study, we observed that ISG15 was significantly up‐regulated in DKD kidney tissues from DKD patients and TECs cultured under HG conditions. Knockdown of ISG15 not only mitigated fibrosis and inflammation, but also improved renal function in DKD mice. In addition, ISG15 knockdown ameliorated HG‐induced TECs fibrosis and inflammation, suggesting ISG15 as a promising therapeutic approach against fibrosis and inflammation in DKD.

Programmed cell death, such as pyroptosis regulated by gasdermin family proteins, may contribute to the parenchymal cell loss in kidney disease and are implicated in the regulation of fibrosis and inflammation.^40^, ^41^, ^42^ Growing evidence showed that NLRP3 and its role in pyroptosis have been implicated in failure of multiple tissues, such as the heart, liver and kidney.^43^, ^44^, ^45^ Studies have revealed the GSDMD as a key downstream effector of inflammatory caspases‐mediated pyroptosis in the kidney. Increased pyroptosis of renal parenchymal cells as a pathologic cause of acute and chronic kidney diseases. This study revealed that inhibiting pyroptosis by reducing GSDMD expression alleviated fibrosis and inflammation in the kidney of DKD mice. Additionally, ISG15‐mediated pyroptosis could promote ongoing tissue fibrosis and inflammation, implicating ISG15‐mediated pyroptosis as a potential amplification mechanism in DKD progression.

Mitochondria are important regulators of cellular energy metabolism and cellular redox homeostasis. In DKD, there is a significant increase in mitochondrial damage characterised by elevated mtROS levels and mtDNA depletion. Persistent mitochondrial injury is closely correlated with the severity of renal fibrosis, inflammation.^46^ Due to high energy requirements of the proximal tubule enriching in mitochondria, we investigated the potential involvement of mitochondria in the damage to TECs regulated by ISG15. Western blot analysis of the cytosolic and mitochondrial subcellular fractions further demonstrated that ISG15 was enriched in the mitochondria of TECs. In our study, we confirmed that ISG15 disrupted mitochondrial integrity and function, and knockdown of ISG15 significantly attenuated mtROS production and mtDNA leakage, indicating a diverse role of ISG15 in the regulation of mitochondrial functions.

The cGAS–STING pathway, which recognises cytoplasmic nucleic acids and initiates an immune response through the downstream signalling involving transcription factors IRF3 and NF‐κB, has been identified as a critical regulator of cancer due to its involvement in inflammatory processes and immune regulation.^47^, ^48^ Our study reveals significant activation of the cGAS–STING pathway in both diabetic kidney tissues and high glucose‐stimulated TECs, which drives pyrpoptosis and promotes fibrotic progression. Inhibition of STING weakened this effect. Also, we found that STING, acting as an upstream of ISG15, could increase the expression of ISG15, while ISG15 reciprocally modulates STING levels via ISGylation. This revealed a regulatory loop between ISG15 and STING that maintains their overexpression in TECs, indicating that the ISG15–STING regulatory loop may offer a viable treatment option for DKD.

Indeed, there are several experimental limitations and unsolved questions in this work. First, the use of systemic knockout mice in our experiments introduces a caveat: since we cannot rule out that ISG15 deficiency in other cell types might have influenced the observed outcomes. Although our in vitro studies have clearly shown that ISG15 is involved in modulating injury to TECs under HG conditions, it is plausible that ISG15 could also impact other renal cell types as well. Future research should aim to expand on these findings to uncover ISG15's role in a broader range of kidney cell types. Furthermore, the siRNA used in our experiments may potentially exhibit off‐target effects, indiscriminately silencing genes other than the intended targets. This could lead to unforeseen side effects that might complicate the interpretation of our results. Additionally, the precise mechanism by which ISG15 translocates to the mitochondria remains unclear. Future investigations may delve into the target proteins of ISGylation and the molecular mechanisms governing ISG15's mitochondrial translocation. Thereby we can enhance our comprehension of ISG15's role in renal pathophysiology and potentially uncover new therapeutic targets for the treatment of DKD.

In recent years, a variety of post‐translational modifications have been implicated in the pathogenesis of DKD, including phosphorylation, ubiquitination, acetylation, methylation and SUMOylation.^49^, ^50^, ^51^, ^52^, ^53^ Expanding on these findings, our study now provides compelling evidence to include ISGylation within this roster. ISGylation, a post‐translational modification known for its association with fibrotic and inflammatory responses, is a key driver of DKD pathogenesis, particularly in the context of renal tubular injury. Reflecting ISG15's likely pivotal role in DKD progression, our findings suggest that future research could explore ISG15 or its related pathways as promising therapeutic targets. By targeting the ISGylation pathway, it may be possible to mitigate the renal damage associated with diabetes and improve patient outcomes.

Collectively, our data revealed that ISG15 up‐regulation plays a key role in mediating HG‐induced TEC injury and advancing DKD pathogenesis. Furthermore, our findings highlighted the ISG15–mtDNA–STING axis as a crucial hub that integrates pyroptosis, fibrosis and inflammation, emphasising its critical involvement in the development of DKD. In summary, ISG15, given its multifaceted nature, emerges as a promising therapeutic target, including the development of pharmacological interventions and immunomodulation, thereby presenting new avenues for the management of DKD.

## AUTHOR CONTRIBUTIONS

T. L. Q. and Y. S. M. participated in research design. H. L. Z., S. Y. W., D. S. J. and C. X. Y. conducted experiments. H. L. Z., S. Y. W., D. S. J., C. X. Y., W. C., C. J. Q. and X. Y. S. performed data analysis. T. L. Q., Y. S. M., H. L. Z., S. Y. W. and D. S. J. contributed to the writing of the manuscript. All authors have read and approved the final manuscript.

## CONFLICT OF INTEREST STATEMENT

The authors declare no conflicts of interest.

## ETHICS STATEMENT

This animal experiment was carried out in strict accordance with the principles of the Declaration of Helsinki and the regulations of the Animal Ethics Committee of the First Affiliated Hospital of University of Science and Technology of China (2022‐N (A)‐012).

## Supporting information



Supporting Information

## Data Availability

All data utilised in this work could be obtained from the corresponding author upon reasonable request.
