# Supplementary material for: Elevation of ISG15 promotes diabetic kidney disease by modulating renal tubular epithelial cell pyroptosis
Source: Clin Transl Med. 2025 Jun 3;15(6):e70337. doi: 10.1002/ctm2.70337 (PMC12134392; doi:10.1002/ctm2.70337)
Supplement: Supplementary file 1 — Supporting Information [file CTM2-15-e70337-s001.pdf]

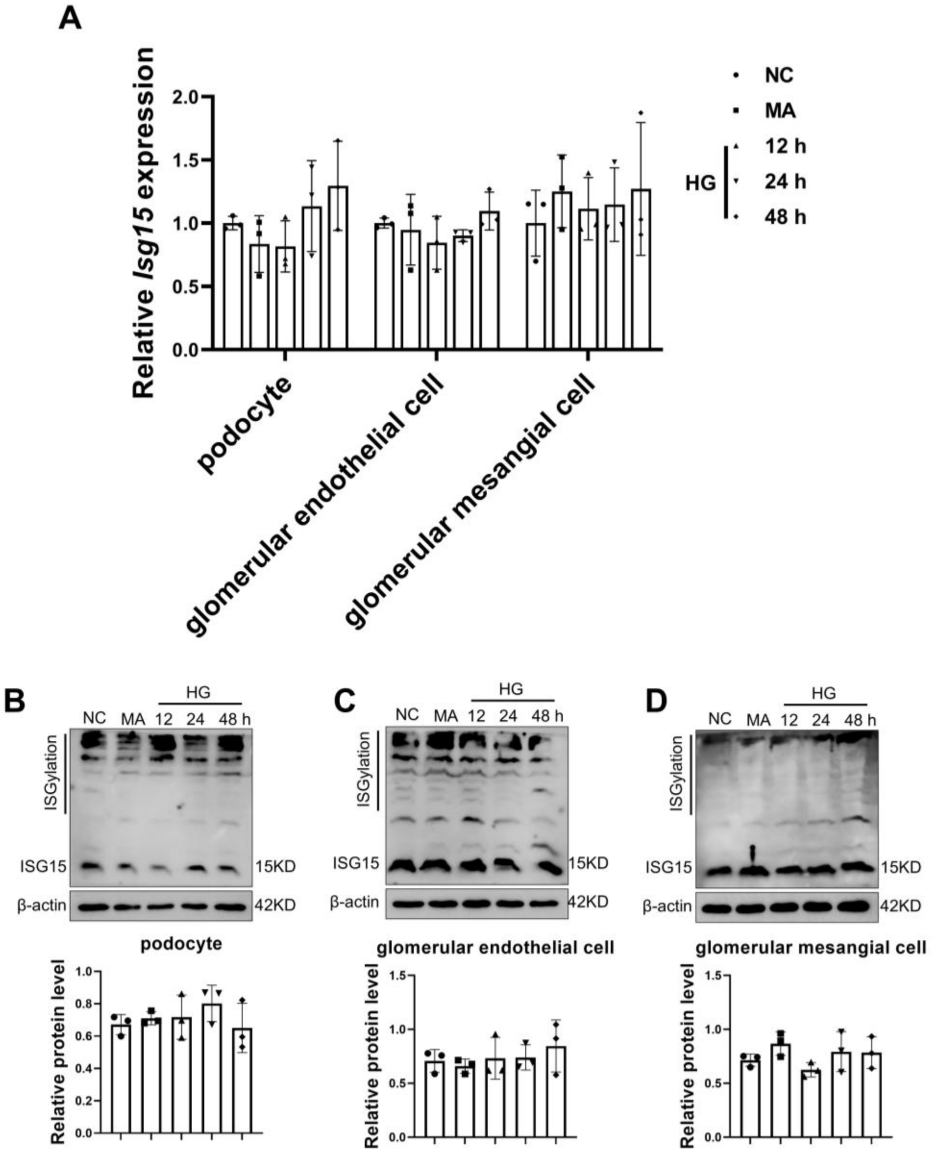

Supplementary Figure 1. ISG15 expression remains unaltered in podocytes, mesangial cells, and endothelial cells under HG stimulation. (A) The expression of *Isg15* was measured in podocyte, glomerular mesangial cell, and glomerular endothelial cell via qPCR (n = 3). (B-D) Western blot analysis ISG15 expression in podocyte (B), glomerular endothelial cell (C), glomerular mesangial cell (D) (n = 3). Results are expressed as the mean ± SD. *$P < 0.05$; **$P < 0.01$; ***$P < 0.001$.

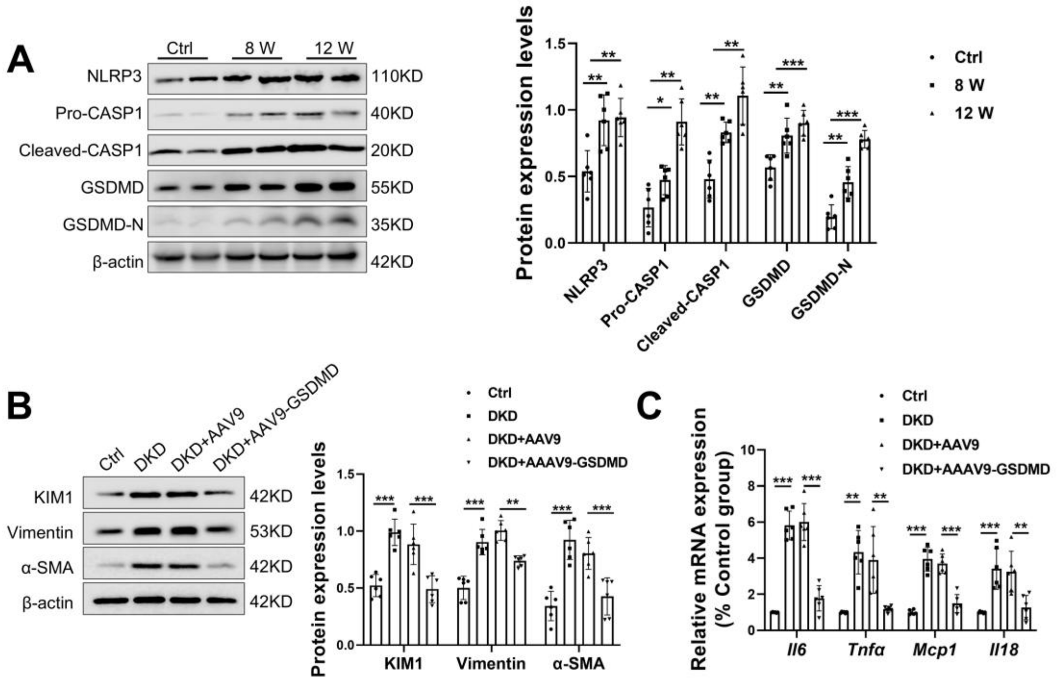

Supplementary Figure 2. Inhibition of pyroptosis blocked renal damage and fibrosis. (A) Western blot analysis and densitometric quantification of pyroptosis-related proteins (NLRP3, Pro-CASP1, Cleaved-CASP1, GSDMD, GSDMD-N) expression in kidney tissues from WT and STZ/HFD-induced DKD mice (n = 6). (B) Western blot analysis and densitometric quantification of KIM1, α-SMA, and Vimentin expression in kidney tissues (n = 6). (C) Relative mRNA level of pro-inflammatory factors (*Il6*, *Tnfa*, *Mcp1*, and *Il18*) in the kidney tissues from WT and STZ/HFD-induced DKD mice, with or without AAV9-ISG15 treatment (n = 6). Results are expressed as the mean ± SD. *$P < 0.05$; **$P < 0.01$; ***$P < 0.001$.

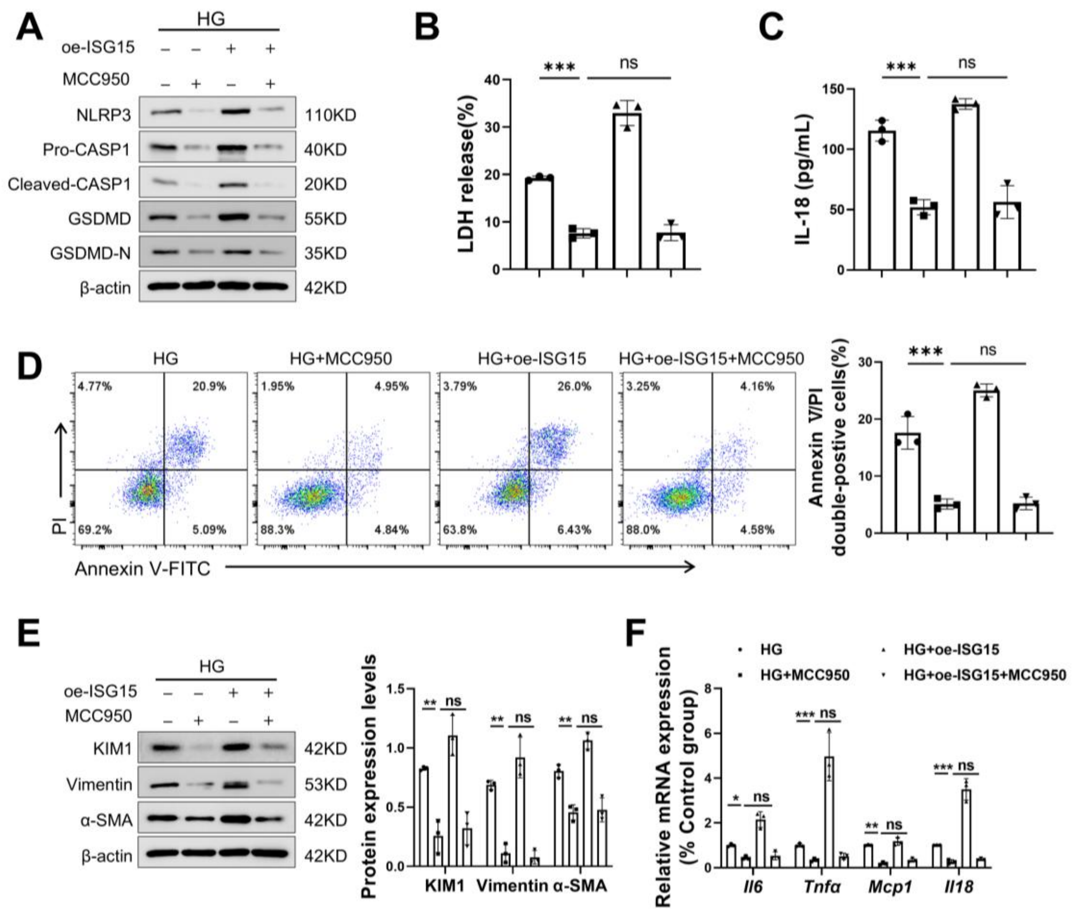

Supplementary Figure 3. ISG15 promoted TECs pyroptosis via NLRP3. (A) Western blot analysis and densitometric quantification of pyroptosis-related proteins (NLRP3, Pro-CASP1, Cleaved-CASP1, GSDMD, GSDMD-N) expression in TECs (n = 3). (B-C) Levels of LDH (B), IL-18 (C) in TECs (n = 3). (D) Flow cytometry analysis and quantitative data depicting the Annexin V/PI double-positive cells rate (n = 3). (E) Western blot analysis and densitometric quantification of KIM1, α-SMA, and Vimentin expression in TECs (n = 3). (F) Relative mRNA level of pro-inflammatory factors (*Il6*, *Tnfa*, *Mcp1* and *Il18*) in TECs (n = 3). TECs were transfected with MCC950 (2 μM) or oe-ISG15 (4 μg), and then cultured in HG medium for 48 h. Results are expressed as the mean ± SD. *$P < 0.05$; **$P < 0.01$; ***$P < 0.001$; ns, not significant.

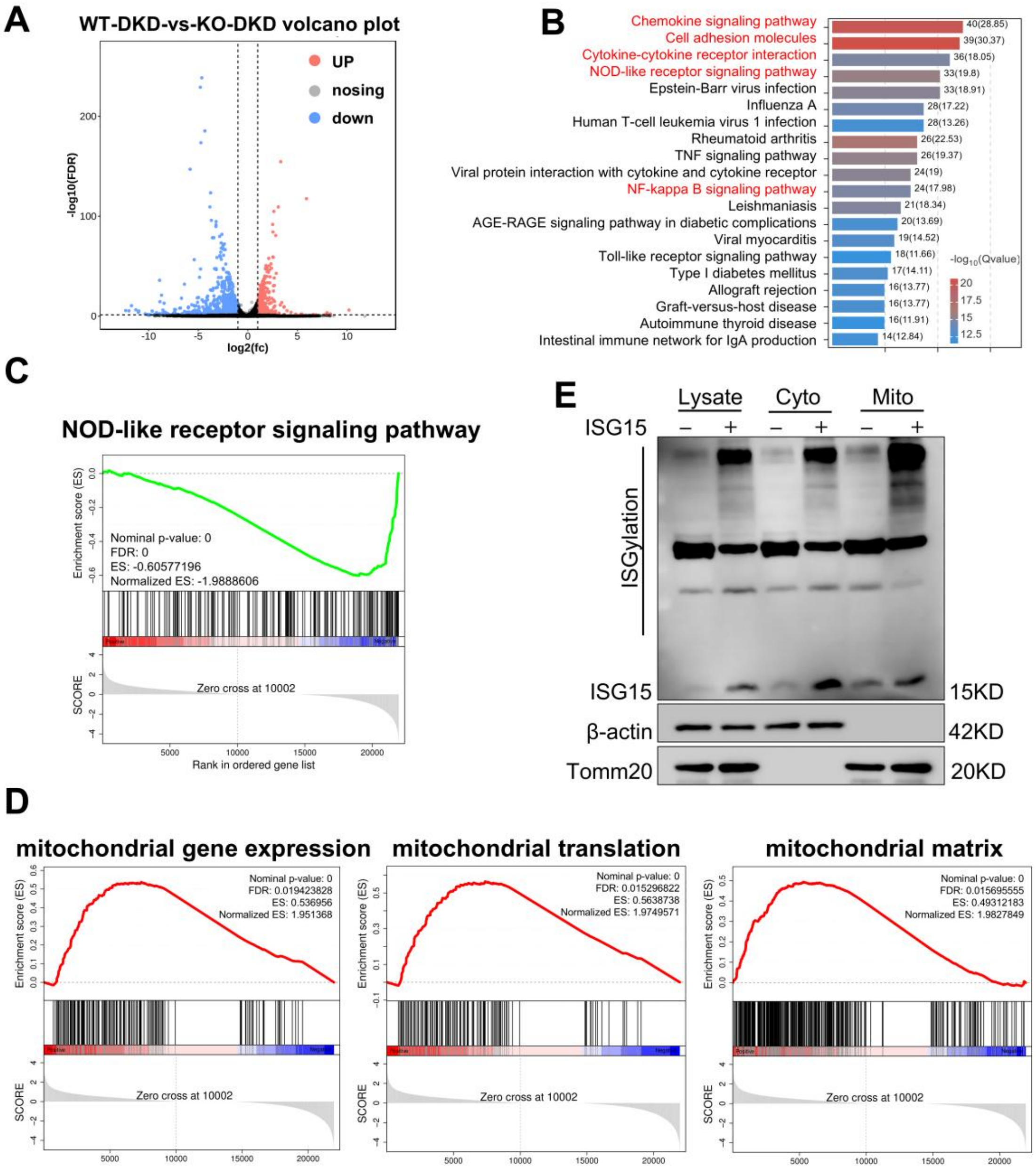

Supplementary Figure 4. ISG15 was involved in mitochondrial impairment. (A-D) RNAseq data analysis of kidney tissues from WT and KO mice with STZ-induced DKD (n = 3). (A) Volcano plot showed the DEGs between WT and KO mice. (B) KEGG pathway analysis for the downregulated genes. (C) GSEA analysis of the NOD-like receptor signaling pathway for downregulated genes. (D) GSEA analysis of the mitochondrial gene expression, mitochondrial translation, and mitochondrial matrix. (E) Western blot analysis ISG15/ISGylation expression in ISG15-overexpressed TECs (n = 3).

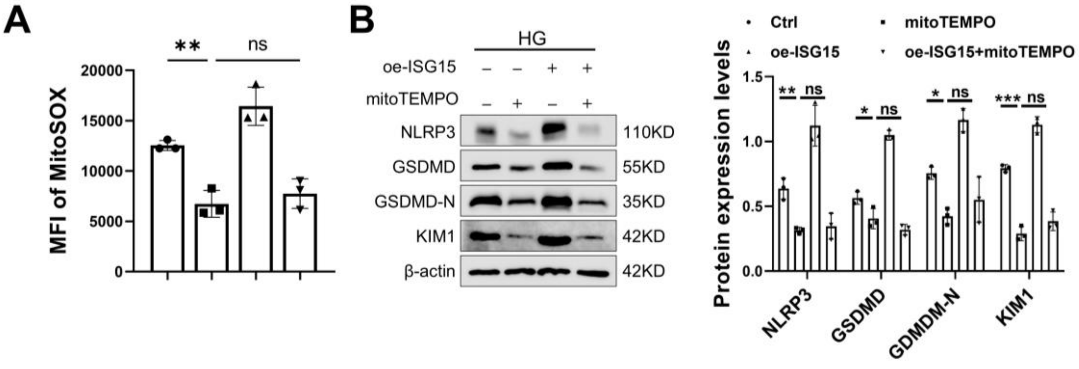

Supplementary Figure 5. Inhibition of mtROS blocked TECs damage induced by ISG15. (A) Flow cytometry analysis and quantitative data depicting the mtROS (n = 3). (B) Western blot analysis and densitometric quantification of NLRP3, GSDMD, GSDMD-N and KIM1 expression in TECs (n = 3). TECs were transfected with MitoTEMPO (25 μM) or oe-ISG15 (4 μg), and then cultured in HG medium for 48 h. Results are expressed as the mean ± SD. *P < 0.05; **P < 0.01; ***P < 0.001; ns, not significant.

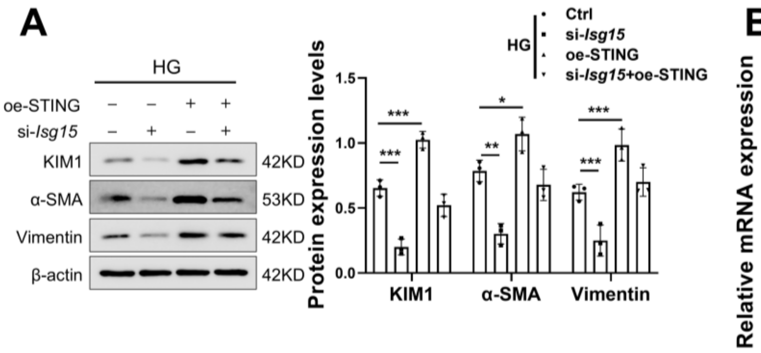

Supplementary Figure 6. ISG15-STING loop promoted TECs fibrosis and inflammation. (A) Western blot analysis and densitometric quantification of KIM1, α-SMA, and Vimentin expression in TECs (n = 3). (B) Relative mRNA level of pro-inflammatory factors (*Il6*, *Tnfa*, *Mcp1* and *Il18*) in TECs (n = 3). TECs were transfected with si-*Isg15* (50 nM) or oe-STING (4 μg), and then cultured in HG medium for 48 h. Results are expressed as the mean ± SD. *P < 0.05; **P < 0.01; ***P < 0.001.

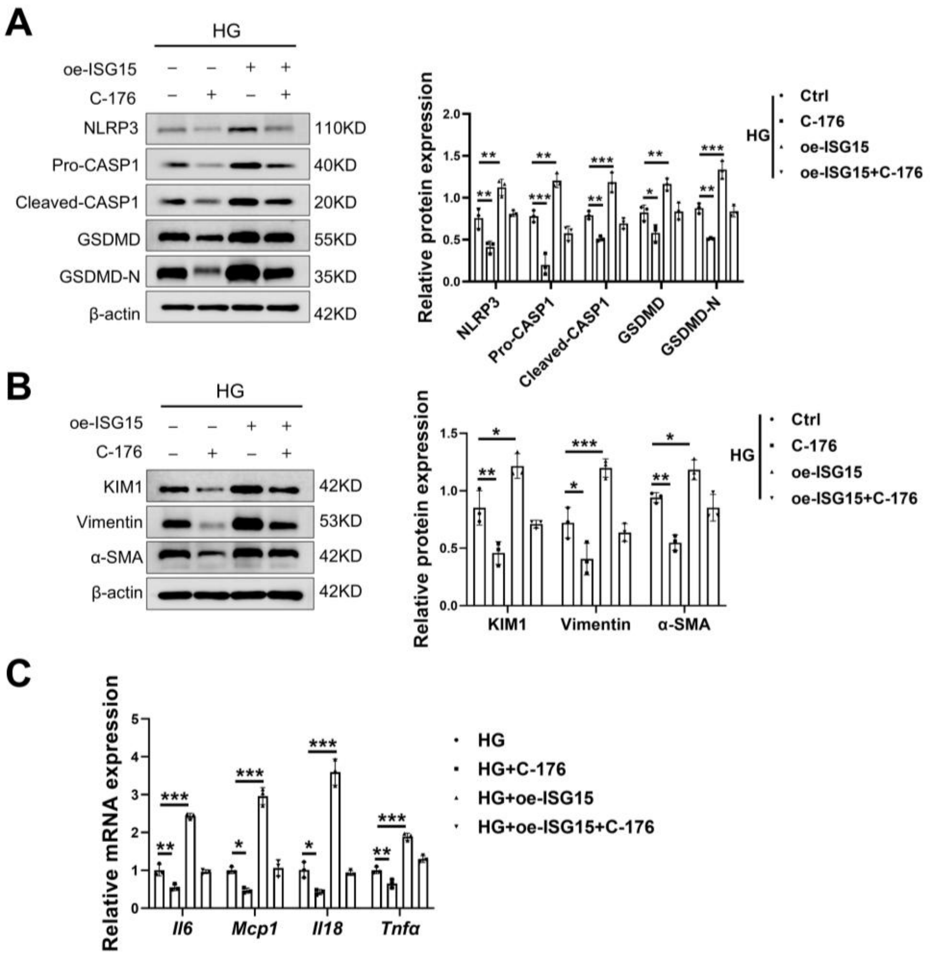

Supplementary Figure 7. Inhibition of STING blocked TECs damage induced by ISG15. (A) Western blot analysis and densitometric quantification of NLRP3, Pro-CASP1, Cleaved-CASP1, GSDMD, GSDMD-N expression in TECs (n = 3). (B) Western blot analysis and densitometric quantification of KIM1, α-SMA, and Vimentin expression in TECs (n = 3). (C) Relative mRNA level of pro-inflammatory factors (*Il6*, *Tnfa*, *Mcp1* and *Il18*) in TECs (n = 3). TECs were transfected with C-176 (10 μM) or oe-ISG15 (4 μg), and then cultured in HG medium for 48 h. Results are expressed as the mean ± SD. *$P < 0.05$; **$P < 0.01$; ***$P < 0.001$.

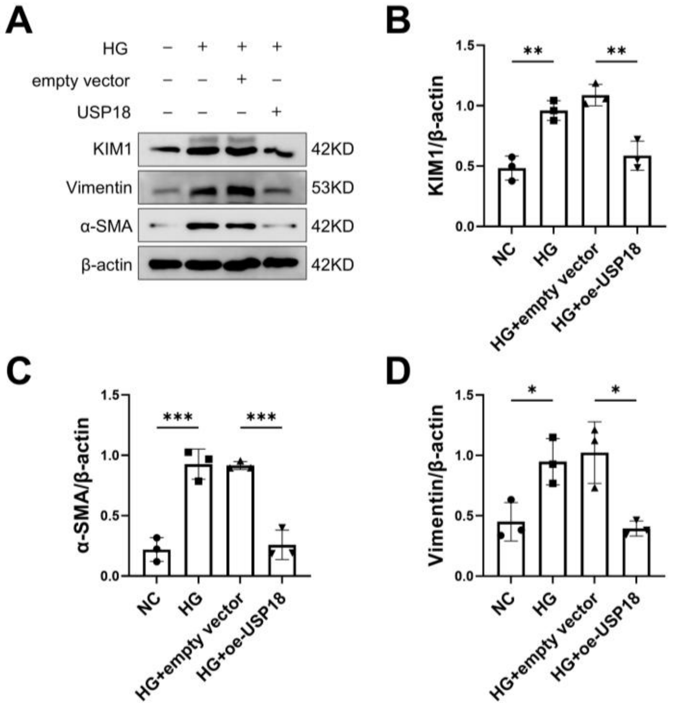

Supplementary Figure 8. Inhibition of ISGylation blocked TEC damage induced by ISG15. (A-D) Western blot analysis and densitometric quantification of KIM1(B), α-SMA(C), and Vimentin(D) expression in TECs (n = 3). TECs were transfected with empty vector or oe-USP18 (4 μg), and then cultured in HG medium for 48 h. Results are expressed as the mean ± SD. *$P < 0.05$; **$P < 0.01$; ***$P < 0.001$.
